# Glycosylation of 6-methylflavone by the strain *Isaria fumosorosea* KCH J2

**DOI:** 10.1371/journal.pone.0184885

**Published:** 2017-10-05

**Authors:** Monika Dymarska, Jakub Grzeszczuk, Monika Urbaniak, Tomasz Janeczko, Elżbieta Pląskowska, Łukasz Stępień, Edyta Kostrzewa-Susłow

**Affiliations:** 1 Department of Chemistry, Faculty of Biotechnology and Food Science, Wrocław University of Environmental and Life Sciences, Wrocław, Poland; 2 Department of Plant Protection, Plant Pathology and Mycology Division, Faculty of Life Sciences and Technology, Wrocław University of Environmental and Life Sciences, Wrocław, Poland; 3 Plant-Microorganism Interaction Team, Department of Pathogen Genetics and Plant Resistance, Institute of Plant Genetics of the Polish Academy of Sciences, Poznań, Poland; Tallinn University of Technology, ESTONIA

## Abstract

Entomopathogenic fungi are known for their ability to carry out glycosylation of flavonoids, which usually results in the improvement of their stability and bioavailability. In this study we used a newly isolated strain of the entomopathogenic filamentous fungus *Isaria fumosorosea* KCH J2 as a biocatalyst. Our aim was to evaluate its ability to carry out the biotransformation of flavonoids and to obtain new flavonoid derivatives. The fungus was isolated from a spider’s carcass and molecularly identified using analysis of the ITS1-ITS2 rDNA sequence. As a result of biotransformation of 6-methylflavone two new products were obtained: 6-methylflavone 8-*O*-β-D-(4”-*O*-methyl)-glucopyranoside and 6-methylflavone 4’-*O*-β-D-(4”-*O*-methyl)-glucopyranoside. Chemical structures of the products were determined based on spectroscopic methods (^1^H NMR, ^13^C NMR, COSY, HMBC, HSQC). Our research allowed us to discover a new species of filamentous fungus capable of carrying out glycosylation reactions and proved that *I*. *fumosorosea* KCH J2 is an effective biocatalyst for glycosylation of flavonoid compounds. For the first time we describe biotransformations of 6-methylflavone and the attachment of the sugar unit to the flavonoid substrate having no hydroxyl group. The possibility of using flavonoid aglycones is often limited by their low bioavailability due to poor solubility in water. The incorporation of a sugar unit improves the physical properties of tested compounds and thus increases the chance of using them as pharmaceuticals.

## Introduction

Flavonoids are the largest group of polyphenols in our diet. Their consumption is associated with reduced mortality due to heart diseases and with inhibition of cognitive decline in the elderly. Studies in rodents have shown that at least some flavonoids (e.g., naringenin, quercetin), which can be absorbed by the bloodstream, can cross the blood-brain barrier [1,2].

In the brain flavonoids act as neuroprotectants. This involves a number of effects including a potential to protect neurons against injury induced by neurotoxins, an ability to suppress neuroinflammation, and the potential to promote memory, learning and cognitive function [3].

The mechanism of action of flavonoids is still unknown, but several flavonoid-binding sites in the brain have been reported, including adenosine receptors, GABA_A_ (γ-aminobutyric acid type A receptor), δ-opioid, nicotinic, estrogen, and testosterone receptors [1,2].

Ai and coworkers have examined the ability of 17 flavonoid compounds to affect the GABA receptors. Among the compounds tested, 6-methylflavone was the most effective inhibitor for the GABA_A_/benzodiazepine receptors in rat and human *in vitro* models [4].

GABA_A_ receptors are the main inhibitory neurotransmitter receptors of the adult central nervous system. Many neuroactive drugs (anxiolytic, anticonvulsant, muscle relaxant, and sedative-hypnotic benzodiazepines (BZDs)) exert their pharmacological effects by interacting with the γ- aminobutyric acid_A_ (GABA_A_) receptors. Chemical compounds do not interact directly with the GABA sites, but bind to allosteric sites within the GABA_A_ receptor complex. Many chemically diverse groups of compounds can also bind to these sites with high affinity. The BZD receptors may be a recognition site not only for benzodiazepines but also for such compounds as triazolopyridazines, cyclopyrrolones, quinolines, β-carbolines, furanocoumarins, and flavonoids.

GABA_A_ receptors are hetero-oligomeric receptors forming a pentameric structure. At least 16 human GABA_A_ receptor subunits have been described and are classified under six subfamilies of protein subunits.

Many heteropentameric isoforms of the GABA_A_ receptors may be found in the brain based on the fact of the existence of multiple subunits and splicing variants of many of them. Thus it is possible to target drugs to specific GABA_A_ receptor subunit combinations and to achieve more effective action, for example, against epilepsy, anxiety or insomnia [4,5].

Drugs targeting GABA_A_ receptors often show many side effects. Benzodiazepines used in the pharmaceutical industry since the 1960s may disrupt memory, cause clumsiness, enhance the effects of alcohol and barbiturates, and increase the likelihood of developing addictions [5].

6-methylflavone could serve as a natural alternative to some of the traditional psychotropic medications. However, potential application of flavonoid aglycones is limited by their low solubility in organic and aqueous solvents, low permeability through biological membranes and insufficient stability in lipophilic media. The way to overcome these problems may be to attach the sugar unit to the flavonoid aglycone [6,7].

Flavonoid compounds are stored in plants mostly in the form of glycosides. The attachment of a sugar unit has an impact on stability and solubility of flavonoids, and often also determines their bioavailability and bioactivity [8–10].

Flavonoids are used as active compounds in many drugs to treat a wide variety of diseases—from impaired peripheral circulation to radiation poisoning, intoxication and liver diseases [11]. Many of the currently used pharmaceuticals contain flavonoids in the form of glycosides. An example is puerarin (8-*C*-glucoside of daidzein) administered by injection and rutin (quercetin-3-*O*-rutinoside) [12]. In Poland there are over 60 commercially available pharmaceutical preparations containing rutin, among which at least 12 are registered as drugs. Most of them support the immune system and are used in treatment of colds. They are available in the form of tablets (containing up to 500 mg of rutin, usually 25–50 mg), effervescent tablets (50 mg), jelly beans for children (5–8 mg), syrup for children (7–10 mg in 10 mL), or powder for solution for children (15–20 mg). Rutin is also used in preparations strengthening blood vessels in the eyes (10–25 mg per tablet), effervescent tablets to suppress allergy symptoms in children (25 mg), tablets for varicose veins (up to 500 mg), gels and creams for varicose veins, and also in cosmetics [13,14].

The attachment of a sugar unit in plants occurs after the synthesis of a flavonoid aglycone. The reaction is catalyzed by the superfamily of enzymes called UDP-glycosyltransferases (UGTs) with over 90 families, which catalyze the transfer of UDP-activated sugar moieties to specific acceptor molecules [8,9]. The glycosyltransferases are involved in production of biomass, maintain cellular homeostasis by regulating the active levels of hormones, stabilize anthocyanin structures, influence transport of biomolecules by modifying their solubility, and modify bioavailability of plant secondary metabolites [15]. There are two types of glycosyltransferases: Leloir-type and non-Leloir-type. The first group of glycosyltransferases transfers the sugar residue from an activated donor, such as sugar-nucleotide derivatives. Nine such derivatives used by glycosyltransferases in mammals have been identified. In contrast, non-Leloir glycosyltransferases use sugar mono- and diphosphates and glycosylated isoprenoid mono- and diphosphates as donors of a glycosyl residue.

Acceptors of sugar units comprise a wide range of compounds, such as proteins, lipids, and xenobiotics. In the case of flavonoids the nucleophilic oxygen atom from a hydroxyl group acts as the acceptor. The reactions catalyzed by glycosyltransferases are regio- and stereoselective. They may proceed either with retention or inversion of the configuration at the anomeric carbon atom of the substrate [15]. The least numerous group of glycosyltransferases that are capable of glycosylation of flavonoids comprises microorganisms’ enzymes, such as BcGT-1 from *Bacillus cereus*, DSM-13 and YjiC from *Bacillus licheniformis*, OleD from *Streptomyces antibioticus* and XcGT-2 from *Xanthomonas campestris* [9,10].

It is often impossible to obtain flavonoid glycosides by chemical synthesis. Harsh reaction conditions lead to decomposition of aglycones, the yields are low, unwanted anomers are formed and recovery of unreacted substrate is usually impossible, whereas enzymatic glycosylation can help to overcome the steric hindrance [16].

There are literature reports on enzymatic synthesis of flavonoid glycosides. The use of BcGT-1 glycosyltransferase from *Bacillus cereus* allowed glycosides to be obtained from six flavonoid aglycones. The attachment of the glycosyl group most often took place at the C-3 position, but also at C-7 when C-3 was not available. The *B*. *cereus* genome is fully sequenced, and it is known that these bacteria produce four different glycosyltransferases. In the first stage of the experiment the BcGT-1 sequence was amplified by PCR. Then the gene was cloned into *Escherichia coli*. The obtained recombinant BcGT-1 protein was purified using a HiTrap Chelating HP column [17].

Apigenin was the substrate for obtaining C-4’ and C-7 glycosides using UDP-glucosyltransferase YjiC, from *Bacillus licheniformis* DSM 13. As previously, recombinant *E*. *coli* was used to obtain the enzyme. The enzyme was isolated using a His-TALON Gravity Column. Fractions containing purified protein were analyzed via gel electrophoresis, concentrated, and then dialyzed [18].

Although the above described methods contribute to better recognition of action of glycosyltransferases, they have limited chances of finding application in industry. The drawback is the necessity to use very expensive UDP-glucose as a sugar donor [17].

By using living microbial cells we can avoid laborious enzymatic preparations and expensive enzyme cofactors [16]. Therefore it is important to look for microorganisms that are able to perform glycosylation of flavonoid substrates.

Entomopathogenic filamentous fungi constitute a specific group of microorganisms, infesting insects and other arthropods. Several members of this group are used in agriculture as an environmentally friendly alternative to chemical pesticides [19,20]

In addition, entomopathogenic filamentous fungi may serve as a source of metabolites which can be used pharmaceutically. An example is fumosorinone produced by the species *Isaria fumosorosea*, which can be used in the treatment of type II diabetes [21].

*Isaria fumosorosea* is commonly found in the soil and also can be isolated from plants or water on every continent except for Antarctica. This filamentous fungus has been isolated from over 40 species of arthropods. Various strains of *I*. *fumosorosea* are successfully used as mycopesticides. In America, Europe and Asia there are several commercially available products based either on *I*. *fumosorosea* alone or in combination with other entomopathogenic species [22].

One of the most important features of entomopathogenic filamentous fungi is their wide enzymatic potential that allows the use of these microorganisms as biocatalysts in biotransformation processes. Biotransformation is an environmentally friendly way to improve properties of compounds, for example to increase their hydrophilicity and thus affect their bioavailability [23].

According to the literature, microbial glycosylation of flavonoid compounds occurs only in the presence of a free hydroxyl group in the substrate. In most cases, tested microorganisms biotransformed flavonoids into corresponding 7-*O*-glycosides and 3-*O*-glycosides [12].

In the present study for the first time an entomopathogenic filamentous fungus of the species *I*. *fumosorosea*, newly isolated from a spider from Wroclaw, was used as a biocatalyst. 6-Methylflavone was used as a substrate to test the ability of the microorganism to produce new flavonoid glycosides.

## Results and discussion

### Molecular identification of *Isaria fumosorosea* KCH J2 strain

The strain of *Isaria fumosorosea* KCH J2 used in this study was genetically identified by determination of the ITS1-ITS2 sequence. One product of 551 bp was obtained using PCR with ITS4-ITS5 primers, and sequenced. The complete sequences of this product indicated 100% identity to the *Isaria fumosorosea* ITS sequences deposited in the GenBank database (accession numbers: KT583211.1, JF792885.1, GU453923.1, FJ177462.1).

### Biotransformations

This study on microbial transformation of 6-methylflavone (**1**) is the continuation of the research on microbial transformation of monosubstituted flavonoids. In our previous studies we used filamentous fungi mostly of the genera *Aspergillus* and *Penicillium* as biocatalysts. We observed mainly the products of hydroxylation, de-esterification, demethylation and dehydrogenation [6,24,25]. In this study, for the first time we employed the strain of entomopathogenic fungus *I*. *fumosorosea* KCH J2 as a biocatalyst.

**Fig 1:**
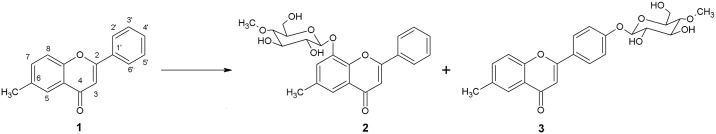
Biotransformations of 6-methylflavone in the culture of *I*. *fumosorosea* KCH J2.

In the course of biotransformation of 6-methylflavone (**1**) in the culture of *I*. *fumosorosea* KCH J2 we observed the formation of two products: **2** and **3** (Fig 1). This is the first report of the introduction of a sugar moiety to a flavonoid compound having no hydroxyl group.

**Fig 2:**
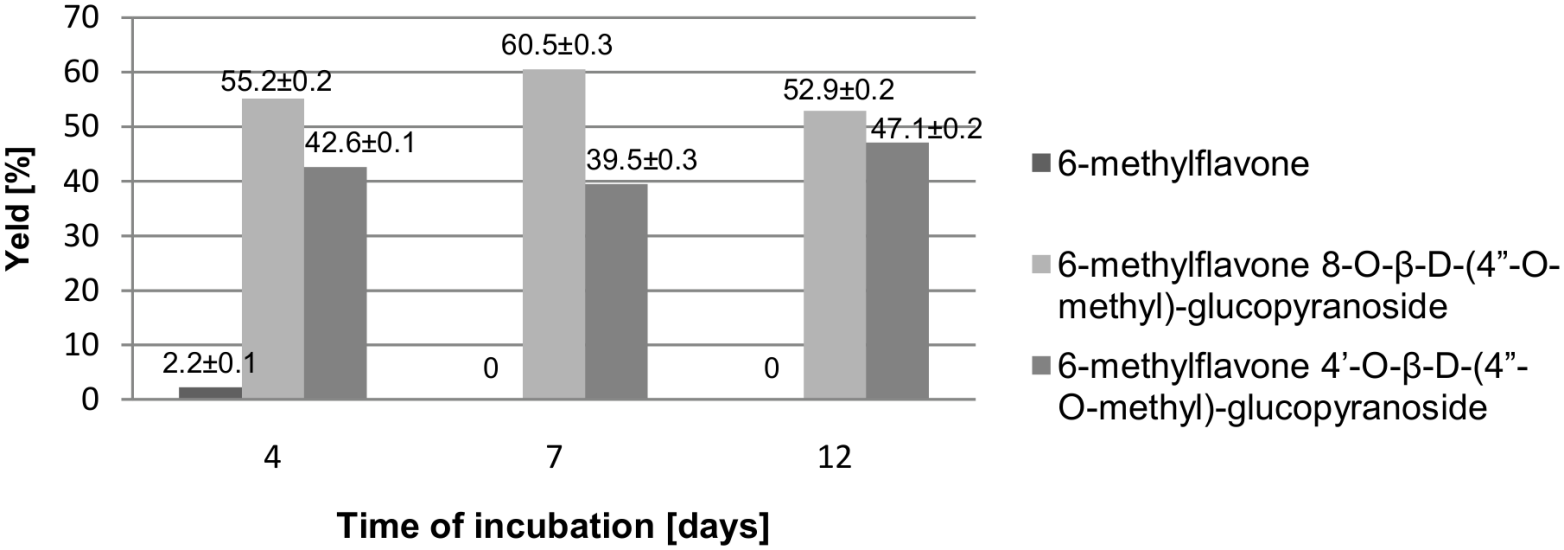
Biotransformations of 6-methylflavone in the culture of *I*. *fumosorosea* KCH J2—Yield [%] of products determined by HPLC.

The reaction was monitored by means of thin-layer chromatography (TLC) and high performance liquid chromatography (HPLC), which provided information about changes in the quantitative ratio of products during the course of the process. The results presented in Fig 2 show the time course of the reaction.

On the 4^th^ day of the initial experiment (screening) we observed both products (**2** and **3**) in the amounts of 55.2% (9.62·10^−4^ mole) and 42.6% (7.44·10^−4^ mole) along with 2.2% (3.77·10^−5^ mole) of the unreacted substrate (**1**) remaining in the reaction mixture. On the 7th day of biotransformation 60.5% (12.98·10^−4^ mole) of product **2**, 39.5% (8.47·10^−4^ mole) of product **3** and no substrate (**1**) was observed. On the 12^th^ day the amounts of products **2** and **3** slightly changed; we observed 52.9% (14.05·10^−4^ mole) of **2** and 47.1% (12.52·10^−4^ mole) of **3**. Prolonging the process over 12 days did not result in any changes in the yields of products. Presented amounts are given in moles per 30 mL of the culture. Our research has not shown the existence of intermediates (hydroxyl or *O*-β-D-glucopyranoside derivatives of 6-methylflavone), but it cannot be ruled out that such intermediates are formed during the reaction. The increase in the amount of products through the successive days of the process, despite the absence of the substrate in the mixture, may indicate the presence of intermediate products.

**Fig 3:**
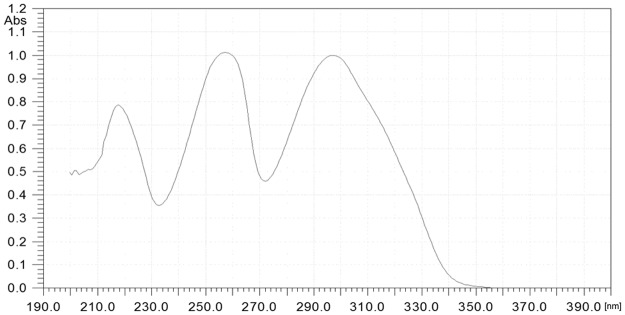
The UV absorption spectrum of 6-methylflavone (1).

**Fig 4:**
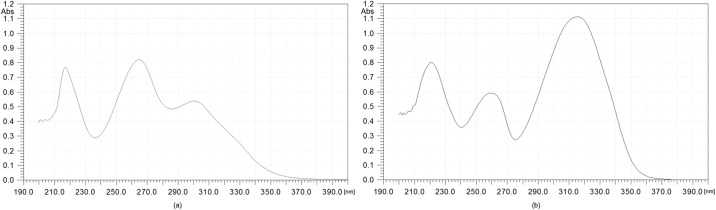
The UV absorption spectra of 6-methylflavone 8-*O*-*β*-D-(4”-*O*-methyl)-glucopyranoside (2) (a) and 6-methylflavone 4’-*O*-*β*-D-(4”-*O*-methyl)-glucopyranoside (3) (b).

Scale-up biotransformation was carried out for a period of 7 days. After this time the products were isolated and purified by means of the preparative TLC method to give 6 mg of product **2** (yield 6.6%) and 12 mg of product **3** (yield 13.2%). The products isolated from the scale-up biotransformation were analyzed by ^1^H NMR and ^13^C NMR spectroscopy, which allowed their structures to be established. They were also used as standards for quantitative analysis carried out by means of HPLC. The UV-absorption spectra for substrate **1** and products **2** and **3** are presented in Figs 3 and 4.

UV-absorption maxima are presented in Table 1.

**Table 1:** UV absorption of 6-methylflavone (1) and its biotransformation products (2 and 3).

Compound	Band I	Band II	Band III
	λ_max_ [nm]	λ_max_ [nm]	λ_max_ [nm]
6-methylflavone (**1**)	298	258	217
8-*O*-β-D-(4”-*O*-methyl)-glucopyranoside (2)	300	264	217
4’-*O*-β-D-(4”-O-methyl)-glucopyranoside (3)	316	259	221

One of the most common methods of identification of flavonoids is UV-visible (UV—Vis) detection. Flavonoids have two characteristic absorption bands: band I (from the B- and C-rings of the flavane nucleus) and band II (from the A-ring) [26]. In the spectrum of product **2** we observed a bathochromic shift of the first absorption band (by 2 nm compared to the substrate), and the second absorption band is also moved towards a longer wavelength (by 6 nm compared to the substrate). In the case of product **3** for the first absorption band we observed a bathochromic shift by 18 nm and for the second one by 1 nm compared to the substrate. When comparing the intensity of the absorption bands with product **1**, the observed hyperchromic effect for the second band in product **2** confirms the presence of a substituent with oxygen atoms in the A ring. Similarly, the hyperchromic effect observed for the first band in product **3** proves the presence of a substituent with oxygen atoms in the B ring [27]. The bathochromic shifts of the maximum absorption bands in products **2** and **3** are due to delocalization of π and n electrons, which facilitate electron excitations associated with n → π* and π → π* transitions [28].

The functionalization of the substrate is confirmed in the ^13^C NMR spectrum by the movement of the C-8 signal from δ = 135.9 ppm to δ = 146.1 ppm for compound **2** and the C-4’ signal from δ = 132.4 to δ = 161.3 ppm for compound **3** –frequencies that are characteristic for aromatic carbons attached to oxygen.

The presence of a glucose unit in molecules **2** and **3** is confirmed by five characteristic carbon signals observed in the region from δ = 80.1 ppm to δ = 62.0 ppm in the ^13^C NMR spectra, along with proton signals of δH ranging from about δ = 3.9 ppm to δ = 3.2 ppm in the ^1^H NMR spectra. Additionally, the attachment of a sugar unit to substrate **1** is confirmed by a one-proton doublet visible at δ = 5.23 ppm in the ^1^H NMR spectrum of **2** and at δ = 5.14 ppm in the spectrum of **3**, which are due to protons at the hemiacetal carbon atoms. A three-proton singlet at δ = 3.62 ppm in the ^1^H NMR and the corresponding signal at δ = 60.6 ppm in the ^13^C NMR prove that one of the hydroxyl groups has been methylated.

The *O*-methylation occurred in the C-4 hydroxyl group of the glucose moiety, which was confirmed in the correlation spectra of compounds **2** and **3**, where the proton signals due to—OCH_3_ (δ = 3.62 ppm) are coupled with the signal of C-4 (δ = 80.1 ppm) in the glucose unit.

In compound **2** the sugar unit is attached to C-8, because in the correlation spectrum the signal due to the proton at the hemiacetal carbon atom (δ = 5.23 ppm) is coupled with the C-8 signal (δ = 147.4 ppm).

In compound **3** the sugar unit is attached to C-4’ of the flavonoid skeleton, because the signal due to the proton at the hemiacetal carbon atom in glucose (δ = 5.14 ppm) is correlated with the signal due to C-4’ (δ = 161.3 ppm).

Chemical shifts of the other signals in the ^1^H and ^13^C NMR spectra have only slightly changed, which indicates that the flavone skeleton remained intact.

In the literature there are reports on biotransformation of flavonoid compounds in cultures of entomopathogenic fungi. The microorganisms used as biocatalysts are primarily fungi of the genus *Beauveria*. Bartmańska et al. described biotransformations of 8-prenylnaringenin in the culture of *Beauveria bassiana* AM278, leading to 7-*O*-*β*-D-glucopyranoside and 7-*O*-*β*-D-4”’-*O*-methylglucopyranoside [29]. Biotransformation of xanthohumol in the culture of the *B*. *bassiana* AM446 strain led to formation of 4’-*O*-*β*-D-(4”’-*O*-methyl)-glucopyranoside [30].

However, to the best of our knowledge there is no information about using fungi of the genus *I*. *fumosorosea* as biocatalysts for transformation of flavonoids.

In the available literature there are no reports on such a one-step process leading to an *O*-methyl sugar derivative of a flavonoid. Schocken et al. in their study on biotransformations of the fungicide cyprodinil used 12 strains of filamentous fungi, ray fungi (actinomycetes) and bacteria. For nine of the tested microorganisms a hydroxyl derivative of the substrate was obtained as a product. *Beauveria bassiana* ATCC 7159 was the only biocatalyst that afforded a product with *O*-methyl glucose attached (yield 80%), and no intermediate hydroxyl derivative was observed [31].

According to the available literature reports there are only microorganisms of the genus *Beauveria* that are capable of attaching *O*-methylglucose to a xenobiotic substrate. There are no detailed studies on kinetics of this process, yet. Yuan et al. carried out biotransformation of (-)-maackiain (pterocarpan) in the culture of *B*. *bassiana*. In the first 12 h of the experiment they observed a glycoside derivative, and later on a *O*-methylglucose derivative appeared in the cultivation medium. In the next stage of the experiment (-)-maackiain glycoside was added to the culture of *B*. *bassiana*, which resulted in obtaining a 4′-methylglucose derivative at a high yield (93%). This leads to the conclusion that 4′-methylglycosylation is a two-step process [32].

## Materials and methods

### Chemicals

The substrate for biotransformations (6-methylflavone) was purchased from Sigma Chemical Company (St. Louis, MO, USA).

#### Spectral data of substrate

6-methylflavone (**1**) C_16_H_12_O_2_ (colorless crystals), R_t_ 20.06 (HPLC);

^1^H NMR (600 MHz) (Acetone-D_6_) δ (ppm): 8.12 (m, 2H, H-2’ and H-6’); 7.94 (s, 1H, H-5); 7.67 (s, 2H, H-7 and H-8); 7.63 (m, 3H, H-3’, H-4’, H-5’); 6.88 (s, 1H, H-3); 2.50 (s, 3H, C6-C*H*_3_).

^13^C NMR (151 MHz, Acetone-D_6_) δ (ppm):178.0 (C-4), 163.8 (C-2), 155.4 (C-8a), 136.1 (C-6), 135.9 (C-8), 132.8 (C-1’), 132.4 (C-4’), 130.0 (C-3’ and C-5’), 127.2 (C-2’ and C-6’), 125.3 (C-5), 124.5 (C-4a), 119.0 (C-7), 107.8 (C-3), 20.9 (C6-CH_3_).

### Microorganism

#### Collection of the material

A corpse of a spider overgrown by a fungus was found in the urban green area of Wrocław (Poland), under a bridge crossing the Odra river, on the structural elements of the platform (51°06'12.1"N 17°04'15.6"E).

The corpse of the spider was carefully collected using sterile forceps and placed in a sterile tube of 50 mL capacity. The obtained material is deposited in the Collection of the Department of Plant Pathology and Mycology in the Department of Plant Protection of the University of Life Sciences in Wroclaw.

#### Propagation of structures of the fungus

The corpse of a spider with visible fungal hyphae was carefully removed from the tube and placed on a sterile Petri dish in a sterile room. Then, using a microbiological needle, fragments of hyphae with visible abundant sporulation were gently collected.

Thus obtained material was placed on a Petri dish (dotted) with PDA medium (BIOCORP, ingredients: potato extract 4 g/L, glucose 20 g/L, agar 15 g/L, distilled water filled to 1 L, acidified with citric acid to a pH of ~5).

The Petri dish was wrapped with Parafilm to prevent the growing culture from potential contamination with microorganisms coming from the air. The Petri dish was then stored at room temperature until fungal sporulation.

Growing mycelium of *Isaria fumosorosea* strain was purified and maintained in pure culture for 7 days on PDA medium for genomic DNA extraction.

#### Genetic identification

Genomic DNA was extracted using the modified CTAB (hexadecyltrimethylammonium bromide) method described previously [33]. The DNA extract was stored at −20°C.

Species identification was based on the Internal Transcribed Spacer ribosomal DNA sequence analysis using the following primers: ITS4, 5'- TCCTCCGCTTATTGATATGC -3' and ITS5, 5'- GGAAGTAAAAGTCGTAACAAGG -3' [34].

PCR was done in 20 μL aliquots using C-1000 thermal cyclers (Bio- Rad, Hercules, CA, USA). Each reaction tube contained 0.4 μL of Phire II HotStart Taq DNA polymerase (Thermo Scientific, Espoo, Finland), 4 μL of 5× PCR buffer, 12.5 pmol of forward/reverse primers, 2.5 mM of each dNTP, and about 20 ng of fungal DNA. PCR conditions were as follows: 30 s at 98°C; 35 cycles of 5 s at 98°C, 5 s at 56°C, and 15 s at 72°C; and 1 min at 72°C. The amplicon was electrophoresed in 1.5% agarose gel (Invitrogen) with Midori Green dye (Nippon Genetics Europe GmbH).

For sequence analysis, the PCR-amplified DNA fragment (551 bp in length) was purified with exonuclease I (Thermo Scientific) and shrimp alkaline phosphatase (Thermo Scientific) using the following program: 30 min at 37°C and 15 min at 80°C. The DNA fragment was labeled using a forward primer and the BigDye Terminator 3.1 kit (Applied Biosystems, Foster City, CA, USA), according to the producer’s recommendations and precipitated with 96% ethanol. Sequence reading was performed using Applied Biosystems equipment. The sequence of the PCR fragment was aligned using the BLASTn algorithm.

### Analysis

The course of the biotransformation was evaluated by chromatographic methods (TLC, HPLC). TLC analysis was carried out using TLC Silica gel 60/Kieselguhr F_254_ plates (Merck, Germany). The developing system was a mixture of chloroform and methanol in the ratio of 9:1. Compounds were visualized using 5% aluminum chloride solution in ethanol. The plates were observed at two wavelengths: 254 and 365 nm.

HPLC analyses were performed with a Waters 2690 instrument equipped with a Waters 996 photodiode array detector, using an ODS 2 column (4.6 x 250 mm, Waters) and a Guard-Pak Inserts μBondapak C18 pre-column. Separation conditions were as follows: gradient elution, using 80% of acetonitrile in 4.5% formic acid solution (eluent A) and 4.5% formic acid (eluent B); flow, 1 mL/min; detection wavelength 280 nm; program: 0–7 min, 10% A 90% B; 7–10 min, 50% A 50% B; 10–13 min, 60% A 40% B; 13–15 min, 70% A 30% B; 15–20 min 80% A 20% B; 20–30 min 90% A 10% B; 30–40 min, 100% A.

Separation of the products obtained by the scale-up biotransformation was achieved using 1000 μm preparative TLC silica gel plates (Analtech, Germany). After development of the chromatograms in chloroform:methanol 9:1, compounds were extracted from the selected gel fragments using ethyl acetate (twice) and tetrahydrofuran (once). The extracts were combined and the solvents were removed using a rotary evaporator.

UV-Vis absorption spectra were obtained in methanol using the Cintra 303 UV-visible spectrometer (GBC Scientific Equipment Ltd.).

NMR analysis was carried out using a Bruker Avance 600 MHz NMR spectrometer with an UltraShield Plus magnet.

### Screening procedure

The microorganism was maintained on potato slants at 4°C. Liquid culture experiments were carried out using Sabouraud medium (1% peptone, 3% glucose). The microorganism was transferred to a 300 mL flask containing 100 mL of the medium. Pre-incubation was carried out on a rotary shaker (140 rpm) at 25°C for 72 hours. Screening was carried out in 100 mL Erlenmeyer flasks containing 30 mL of Sabouraud liquid medium. The pre-grown culture (0.5 mL) was transferred to a flask and after 72-hour incubation, 3 mg of the substrate dissolved in 0.5 mL of tetrahydrofuran was added. The molar concentration of the substrate was 0.42 M. We used a separate flask for culture for each sample collection. The biotransformation was carried out under the same conditions as pre-incubation. After 4, 7 and 12 days of biotransformation the mixtures were extracted with 30 mL of ethyl acetate. The extracts were dried over MgSO_4_ (5 min), concentrated in vacuo and analyzed by TLC. Quantitative analyses of the mixtures were performed by means of HPLC. Calibration curves for quantitative analyses were prepared using isolated and purified biotransformation products as standards.

Stability of the substrate was evaluated under analogous conditions, without using a biocatalyst.

### Scale-up biotransformations

Scale-up biotransformations were carried out in 2 L flasks containing 500 mL of the medium. The pre-incubation culture (1 mL) was transferred to the flask. After 72 hours of incubation, 50 mg of the substrate dissolved in 1 mL of tetrahydrofuran was added. The molar concentration of the substrate was 0.42 M. The scale-up biotransformation was carried out under the same conditions as the screening (140 rpm, 25°C). After complete consumption of the substrate (7 days) metabolites were extracted three times using each time 200 mL of ethyl acetate. The extracts were dried out using MgSO_4_ and concentrated on a rotary evaporator. Product separation was carried out using preparative TLC plates. Product structure was determined by spectroscopic methods (^1^H NMR, ^13^C NMR, COSY, HMBC, HSQC).

### Spectral data of isolated metabolites

6-Methylflavone 8-*O*-*β*-D-(4”-*O*-methyl)-glucopyranoside (**2**): One-week transformation of (**1**) (50 mg) in the culture *I*. *fumosorosea* KCH J2 yielded 6 mg of (**2**) (colorless crystals), *R*_*t*_ = 10.92 min (HPLC);

^1^H NMR (600 MHz) (Acetone-D_6_) δ (ppm): 8.23 (d, 2H, *J*_2’,3’(6’,5’)_ = 7.0 Hz, H-2’ and H-6’); 7.63 (m, 3H, H-3’, H-4’, H-5’); 7.58 (s, 1H, H-5); 7.52 (s, 1H, H-7); 6.90 (s, 1H, H-3); 5.23 (d, 1H, *J* = 6.5 Hz, H-1”); 3.89 (br d, 1H, *J* = 11.9 Hz, H-6”a); 3.76 (dd, 1H, *J* = 11.7, 4.5 Hz, H-6”b); 3.72 (m, 2H, H-2”, H-3”); 3.62 (s, 3H, C-4”-OC*H*_3_); 3.57 (m, 1H, H-5”); 3.32 (m, 1H, H-4”); 2.46 (s, 3H, C6-C*H*_3_).

^13^C NMR (600 MHz, Acetone-D_6_) δ (ppm): 177.8 (C-4), 163.3 (C-2), 147.4 (C-8), 146.1 (C-8a), 135.9 (C-6), 132.7 (C-1’), 132.4 (C-4’), 129.9 (C-3’ and C-5’), 127.4 (C-2’ and C-6’), 125.6 (C-4a), 122.3 (C-7), 118.2 (C-5), 107.5 (C-3), 102.2 (C-1”), 80.1 (C-4”), 78.1 (C-3”), 77.2 (C-5”), 75.0 (C-2”), 61.9 (C-6”), 60.6 (C-4”-OCH_3_), 21.4 (C6-CH_3_).

6-methylflavone 4′-*O*-*β*-D-(4”-*O*-methyl)-glucopyranoside (**3**): One-week transformation of (**1**) (50 mg) in the culture *I*. *fumosorosea* KCH J2 yielded 12 mg of (**3**) (colorless crystals), *R*_*t*_ = 11.22 min (HPLC);

^1^H NMR (600 MHz) (Acetone-D_6_) δ (ppm): 8.06 (d, 2H, *J*_2’,3’(6’,5’)_ = 8.4 Hz, H-2’ and H-6’); 7.93 (s, 1H, H-5); 7.65 (s, 2H, H-7 and H-8); 7.27 (d, 2H, *J*_3’,2’(5’,6’)_ = 8,4 Hz H-3’ and H-5’); 6.79 (s, 1H, H-3); 5.14 (1H, d, *J* = 7.7 Hz, H-1”); 3.91 (d, 1H, *J* = 12.0 Hz, H-6”a); 3.75 (1H, dd, *J* = 11.3, 4.2 Hz, H-6”b); 3.71 (1H, t, *J* = 9.0 H-3”); 3.62 (s, 3H, C-4”-OC*H*_3_); 3.59 (1H, m, H-5”); 3.56 (1H, t, *J* = 8.5 Hz, H-2”); 3,28 (1H, t, *J* = 9.3 Hz, H-4”); 2.50 (1H, s, C6-C*H*_3_).

^13^C NMR (600 MHz, Acetone-D_6_) δ (ppm): 177.9 (C-4), 163.6 (C-2), 161.3 (C-4’), 155.3 (C-9), 135.9 (C-6), 135.7 (C-7), 128.8 (C-2’, C-6’), 126.3 (C-1’), 125.3 (C-5), 124.5 (C-10), 118.9 (C-8), 117.6 (C-3’, C-5’), 106.7 (C-3), 101.2 (C-1”), 80.1 (C-4”), 77.9 (C-3”), 77.2 (C-5”), 74.9 (C-2”), 62.1 (C-6”), 60.6 (C-4”-O*C*H_3_), 20.9 (C6-CH_3_).

## Conclusions

The aim of the present study was to evaluate whether the newly isolated entomopathogenic fungus is capable of transforming flavonoid aglycones into their *O*-methylated glycosides. On the basis of our research we found that apart from *B*. *bassiana* known from the literature, other species of entomopathogenic filamentous fungi can also have this unique property.

As a result of using the *I*. *fumosorosea* KCH J2 strain newly isolated from the environment we obtained two new products: 6-methylflavone 8-*O*-*β*-D-(4”-*O*-methyl)-glucopyranoside and 6-methylflavone 4’-*O*-*β*-D-(4”-*O*-methyl)-glucopyranoside.

Flavonoid compounds are the subject of intensive research, which reveals their wide spectrum of biological activity. However, their application in the pharmaceutical industry is considerably limited due to their low bioavailability, resulting from low water solubility. The introduction of the sugar unit increases stability, water solubility and bioavailability of flavonoid compounds. Hence, our research increases the chance for commercial use of 6-methylflavone in future as a natural alternative to benzodiazepines.

It is often impossible to obtain flavonoid glycosides by chemical synthesis, whereas enzymatic methods, despite their high regio- and stereoselectivity, are still too expensive for commercial use.

So far, an improvement of physical properties of flavonoids with methoxy, methyl or amine groups by biotechnological glycosylation has not been possible, due to the lack of a hydroxyl group that would be able to form an *O*-glycoside bond. The biotransformation system presented in this study is highly efficient, relatively cheap, easy to perform and environmentally friendly and may be an effective tool for introducing the sugar moiety also in the case of other flavonoid substrates, leading to a number of novel flavonoid glycosides.

## Supporting information

S1 Fig ^1^H NMR spectrum of 6-methylflavone (1) (Acetone-D_6_, 600 MHz). (PDF)

S2 Fig ^1^H NMR spectrum of 6-methylflavone (1) (Acetone-D_6_, 600 MHz). (PDF)

S3 Fig ^13^C NMR spectrum of 6-methylflavone (1) (Acetone-D_6_, 600 MHz). (PDF)

S4 Fig ^13^C NMR spectrum of 6-methylflavone (1) (Acetone-D_6_, 600 MHz). (PDF)

S5 Fig COSY NMR spectrum of 6-methylflavone (1) (Acetone-D_6_, 600 MHz). (PDF)

S6 Fig HSQC NMR spectrum of 6-methylflavone (1) (Acetone-D_6_, 600 MHz). (PDF)

S7 Fig ^1^H NMR of 6-methylflavone 8-*O*-*β*-D-(4”-*O*-methyl)-glucopyranoside (2) (Acetone-D_6_, 600 MHz). (PDF)

S8 Fig ^1^H NMR of 6-methylflavone 8-*O*-*β*-D-(4”-*O*-methyl)-glucopyranoside (2) (Acetone-D_6_, 600 MHz). (PDF)

S9 Fig ^1^H NMR of 6-methylflavone 8-*O*-*β*-D-(4”-*O*-methyl)-glucopyranoside (2) (Acetone-D_6_, 600 MHz). (PDF)

S10 Fig ^13^C NMR of 6-methylflavone 8-*O*-*β*-D-(4”-*O*-methyl)-glucopyranoside (2) (Acetone-D_6_, 600 MHz). (PDF)

S11 Fig COSY NMR of 6-methylflavone 8-*O*-*β*-D-(4”-*O*-methyl)-glucopyranoside (2) (Acetone-D_6_, 600 MHz). (PDF)

S12 Fig HSQC NMR of 6-methylflavone 8-*O*-*β*-D-(4”-*O*-methyl)-glucopyranoside (2) (Acetone-D_6_, 600 MHz). (PDF)

S13 Fig HMBC NMR of 6-methylflavone 8-*O*-*β*-D-(4”-*O*-methyl)-glucopyranoside (2) (Acetone-D_6_, 600 MHz). (PDF)

S14 Fig ^1^H NMR of 6-methylflavone 4’-*O*-*β*-D-(4”-*O*-methyl)-glucopyranoside (3) (Acetone-D_6_, 600 MHz). (PDF)

S15 Fig ^1^H NMR of 6-methylflavone 4’-*O*-*β*-D-(4”-*O*-methyl)-glucopyranoside (3) (Acetone-D_6_, 600 MHz). (PDF)

S16 Fig ^1^H NMR of 6-methylflavone 4’-*O*-*β*-D-(4”-*O*-methyl)-glucopyranoside (3) (Acetone-D_6_, 600 MHz). (PDF)

S17 Fig ^13^C NMR of 6-methylflavone 4’-*O*-*β*-D-(4”-*O*-methyl)-glucopyranoside (3) (Acetone-D_6_, 600 MHz). (PDF)

S18 Fig COSY NMR of 6-methylflavone 4’-*O*-*β*-D-(4”-*O*-methyl)-glucopyranoside (3) (Acetone-D_6_, 600 MHz). (PDF)

S19 Fig HSQC NMR of 6-methylflavone 4’-*O*-*β*-D-(4”-*O*-methyl)-glucopyranoside (3) (Acetone-D_6_, 600 MHz). (PDF)

S20 Fig HMBC NMR of 6-methylflavone 4’-*O*-*β*-D-(4”-*O*-methyl)-glucopyranoside (3) (Acetone-D_6_, 600 MHz). (PDF)
