# Supplementary figures and images for: Glycosylation of 6-methylflavone by the strain Isaria fumosorosea KCH J2
Source: PLoS One. 2017 Oct 5;12(10):e0184885. doi: 10.1371/journal.pone.0184885 (PMC5628805; doi:10.1371/journal.pone.0184885)

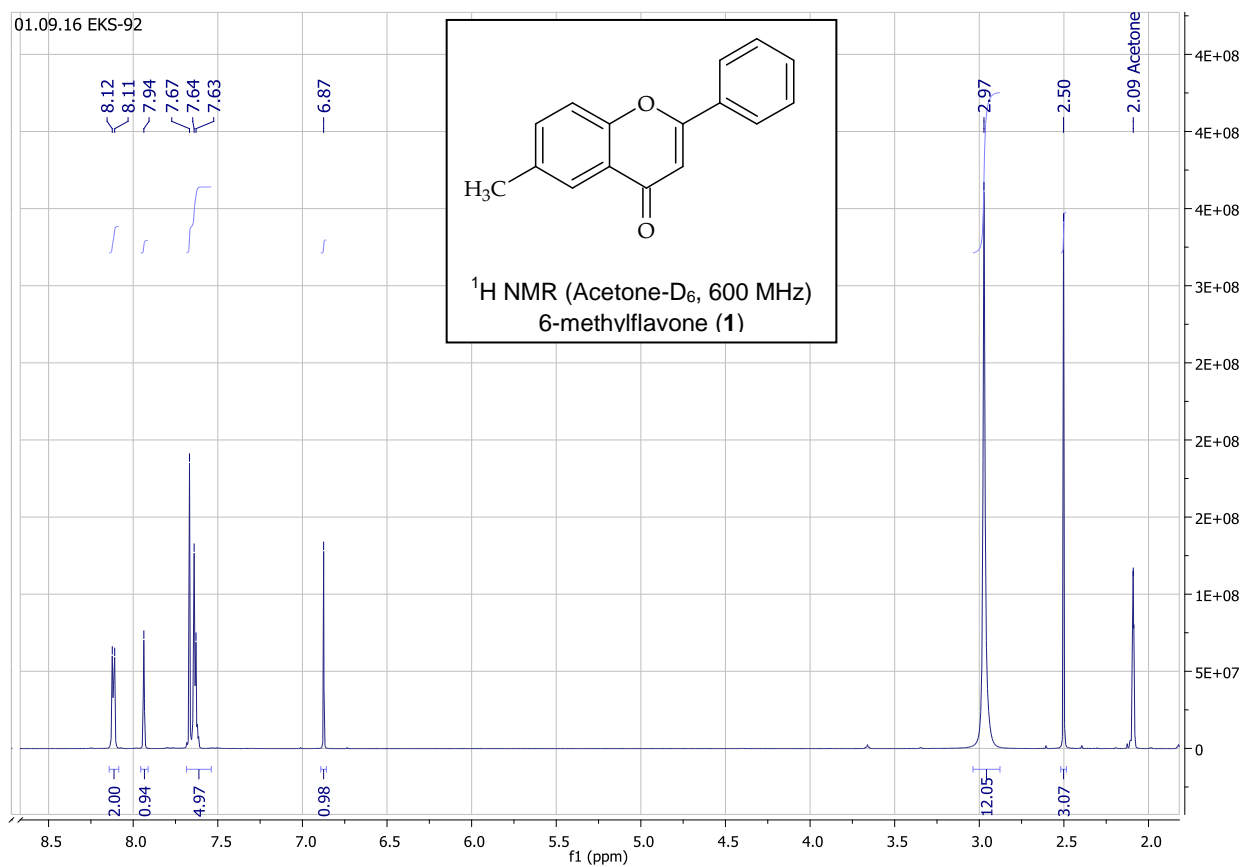

**S1 Fig.  $^1\text{H}$  NMR spectrum of 6-methylflavone (1) (Acetone- $\text{D}_6$ , 600 MHz).**

Supplement: S1 Fig — (PDF) [file pone.0184885.s001.pdf]

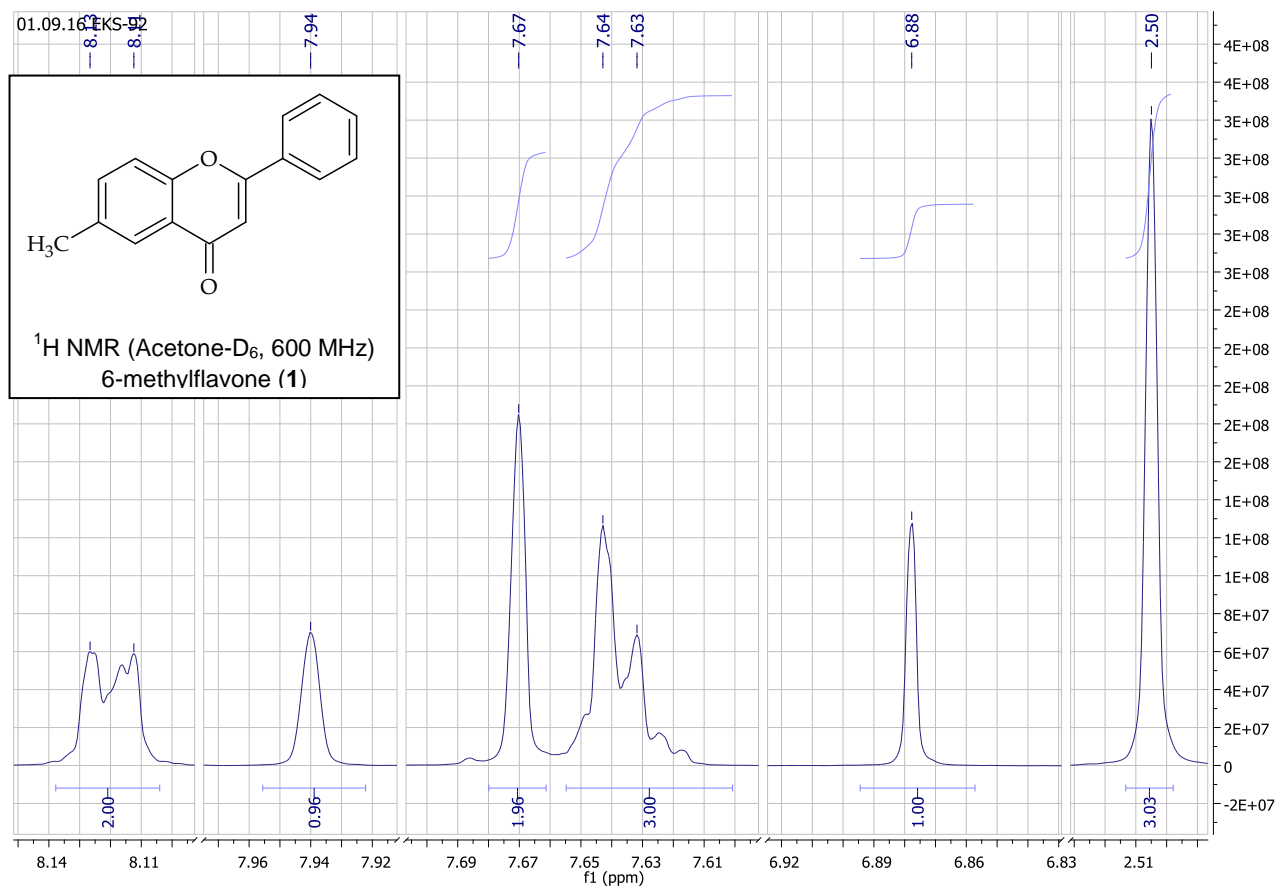

**S2 Fig. <sup>1</sup>H NMR spectrum of 6-methylflavone (1) (Acetone-D<sub>6</sub>, 600 MHz).**

Supplement: S2 Fig — (PDF) [file pone.0184885.s002.pdf]

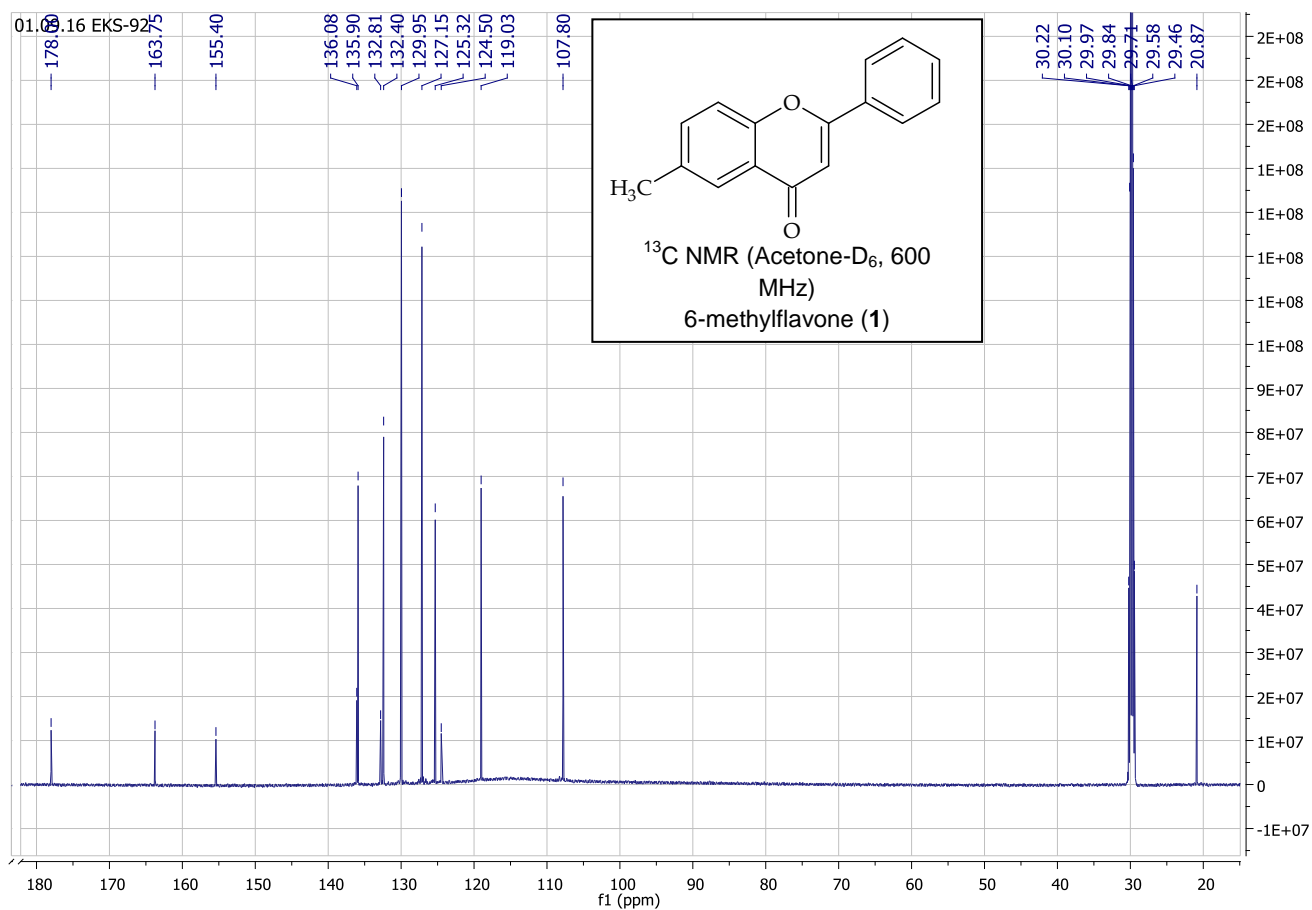

**S3 Fig.**  $^{13}\text{C}$  NMR spectrum of 6-methylflavone (1) (Acetone- $\text{D}_6$ , 600 MHz).

Supplement: S3 Fig — (PDF) [file pone.0184885.s003.pdf]

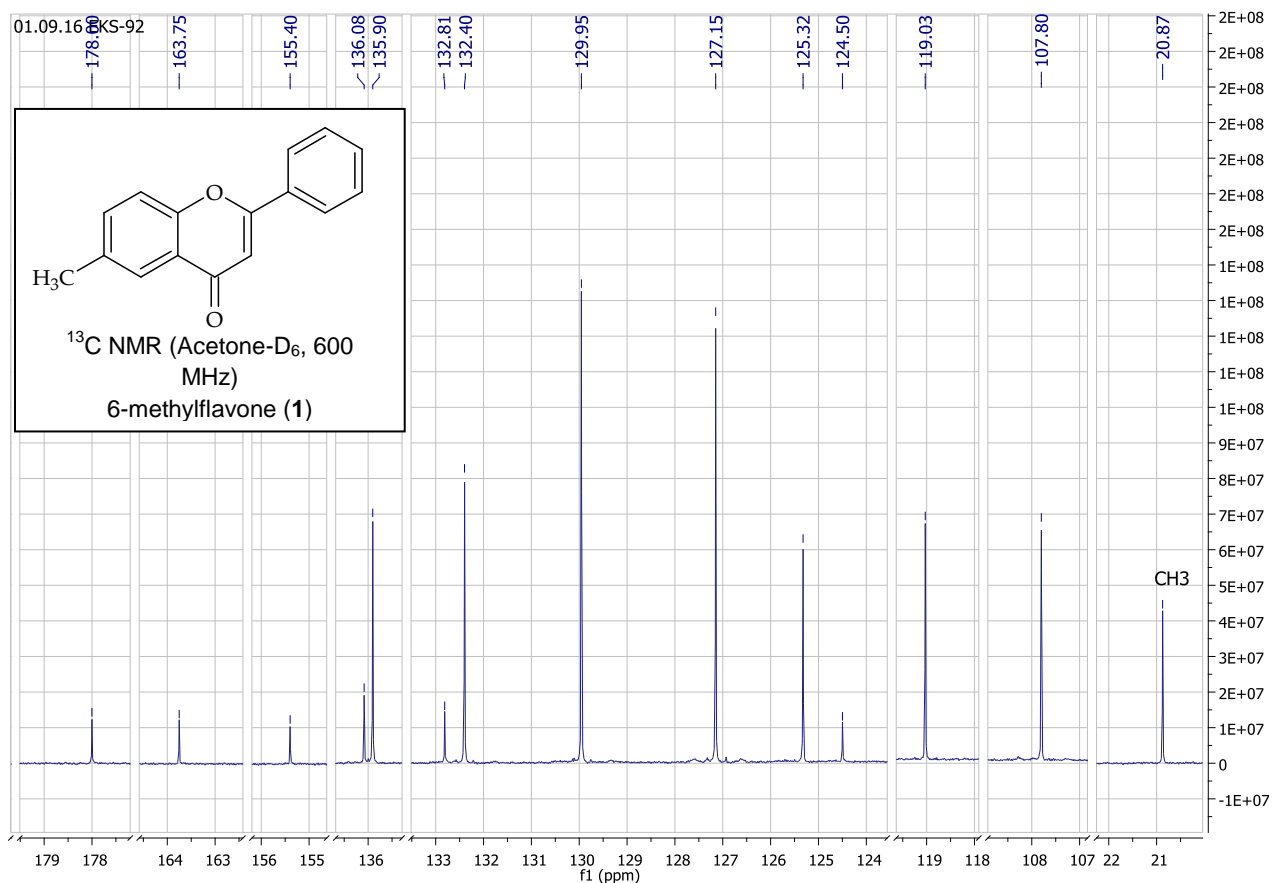

**S4 Fig.** <sup>13</sup>C NMR spectrum of 6-methylflavone (1) (Acetone-D<sub>6</sub>, 600 MHz).

Supplement: S4 Fig — (PDF) [file pone.0184885.s004.pdf]

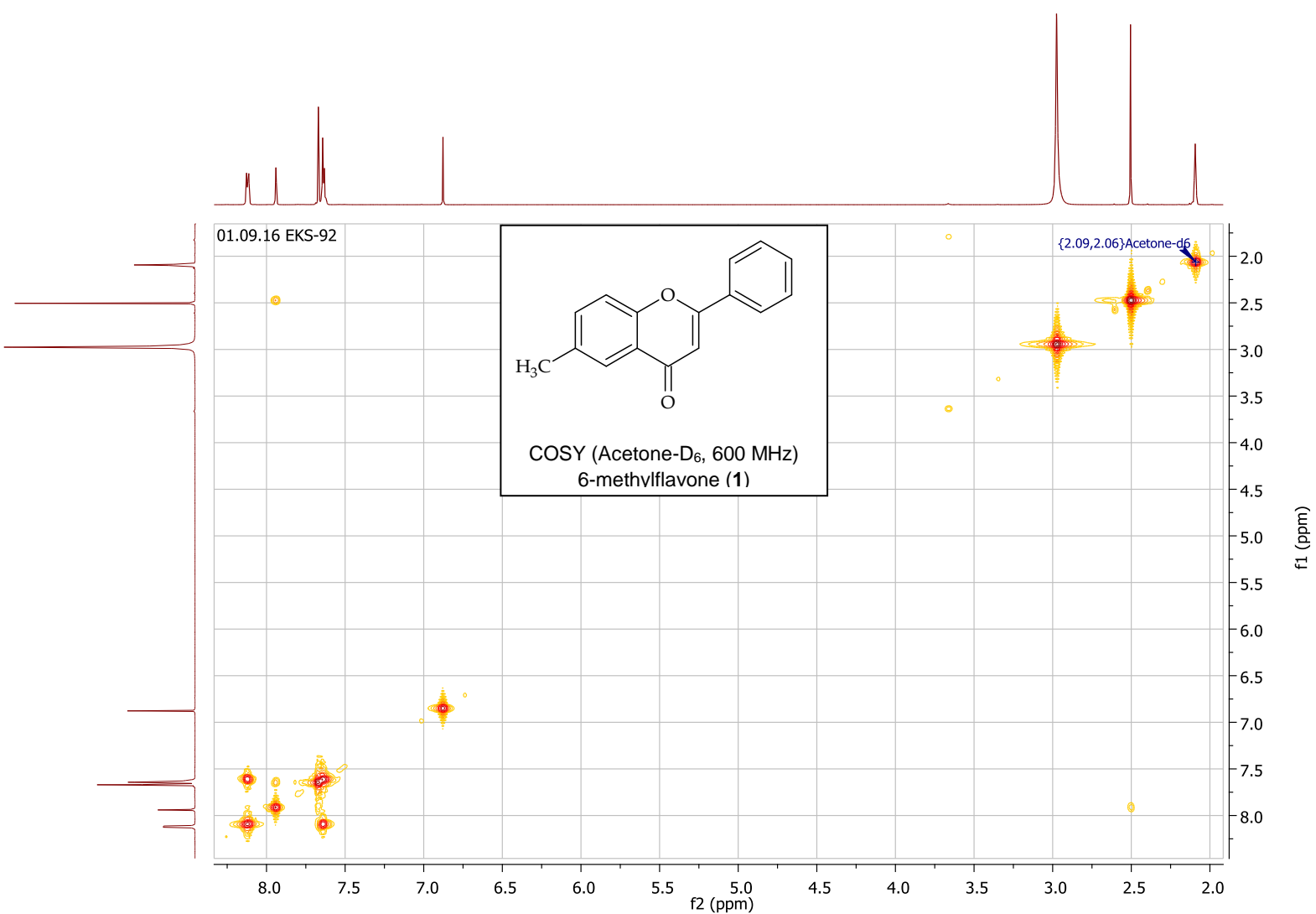

**S5 Fig. COSY NMR spectrum of 6-methylflavone (1) (Acetone-D<sub>6</sub>, 600 MHz).**

Supplement: S5 Fig — (PDF) [file pone.0184885.s005.pdf]

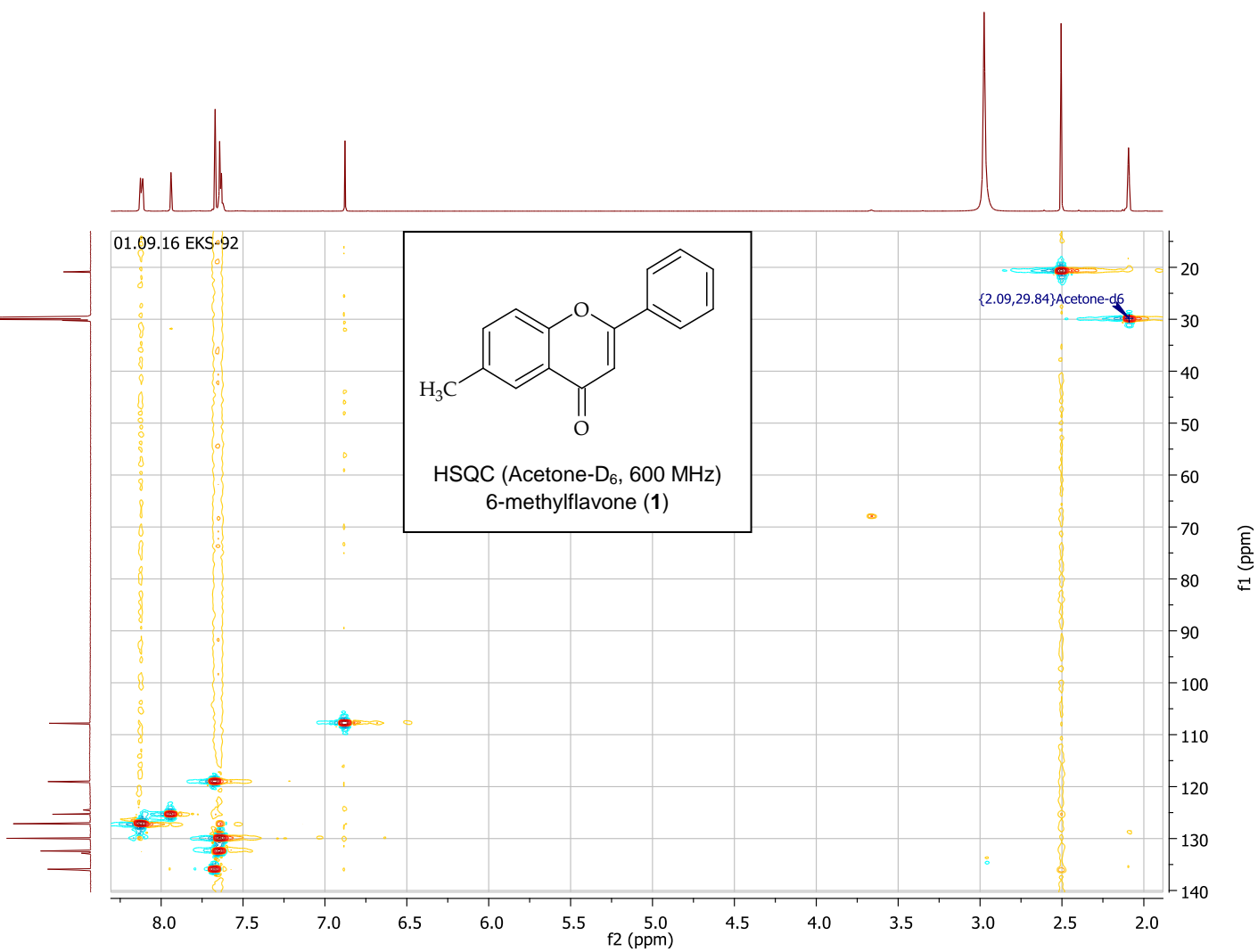

**S6 Fig. HSQC NMR spectrum of 6-methylflavone (1) (Acetone-D<sub>6</sub>, 600 MHz).**

Supplement: S6 Fig — (PDF) [file pone.0184885.s006.pdf]

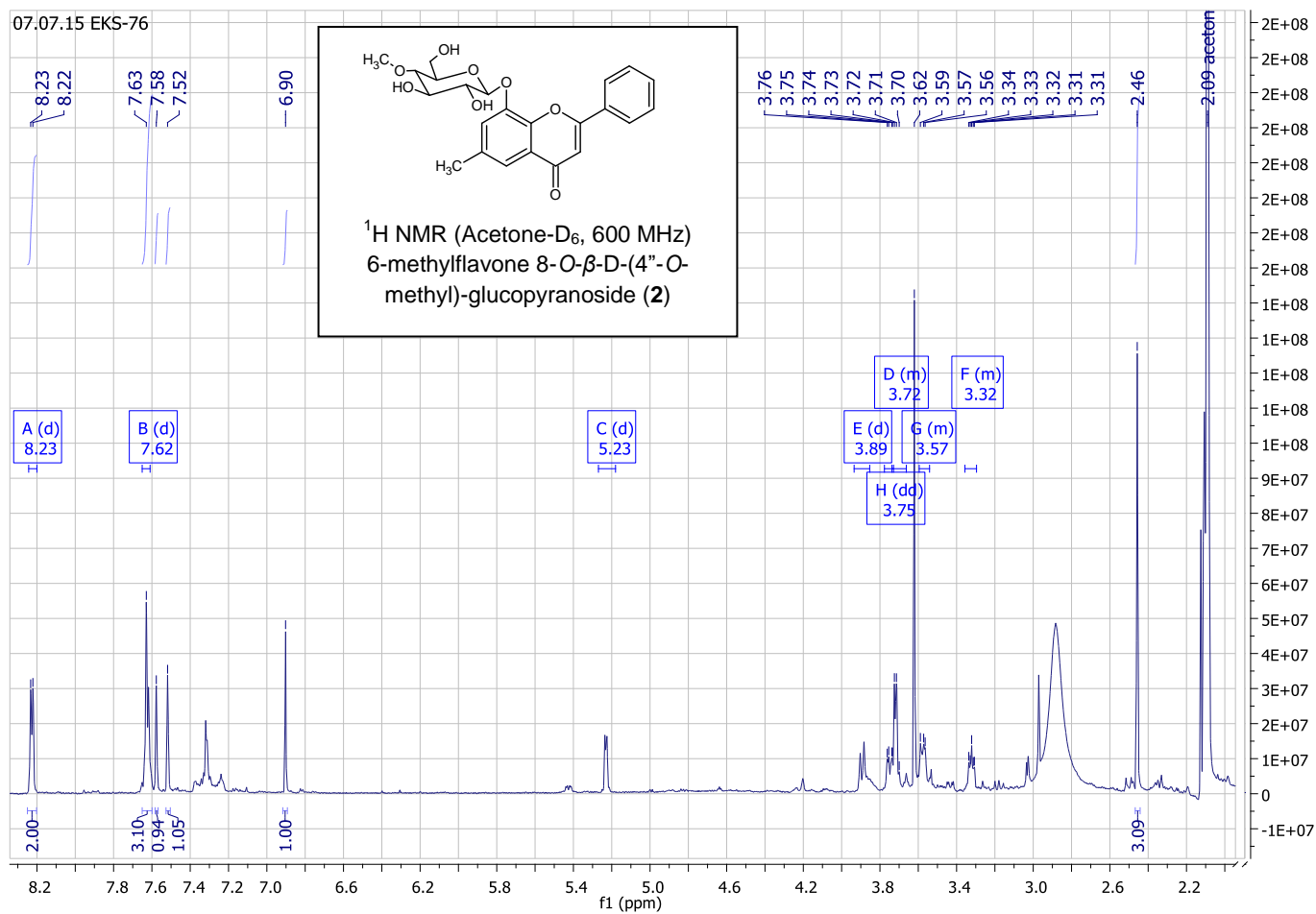

**S7 Fig.**  $^1\text{H}$  NMR of 6-methylflavone 8-O- $\beta$ -D-(4''-O-methyl)-glucopyranoside (2) (Acetone- $\text{D}_6$ , 600 MHz).

Supplement: S7 Fig — (PDF) [file pone.0184885.s007.pdf]

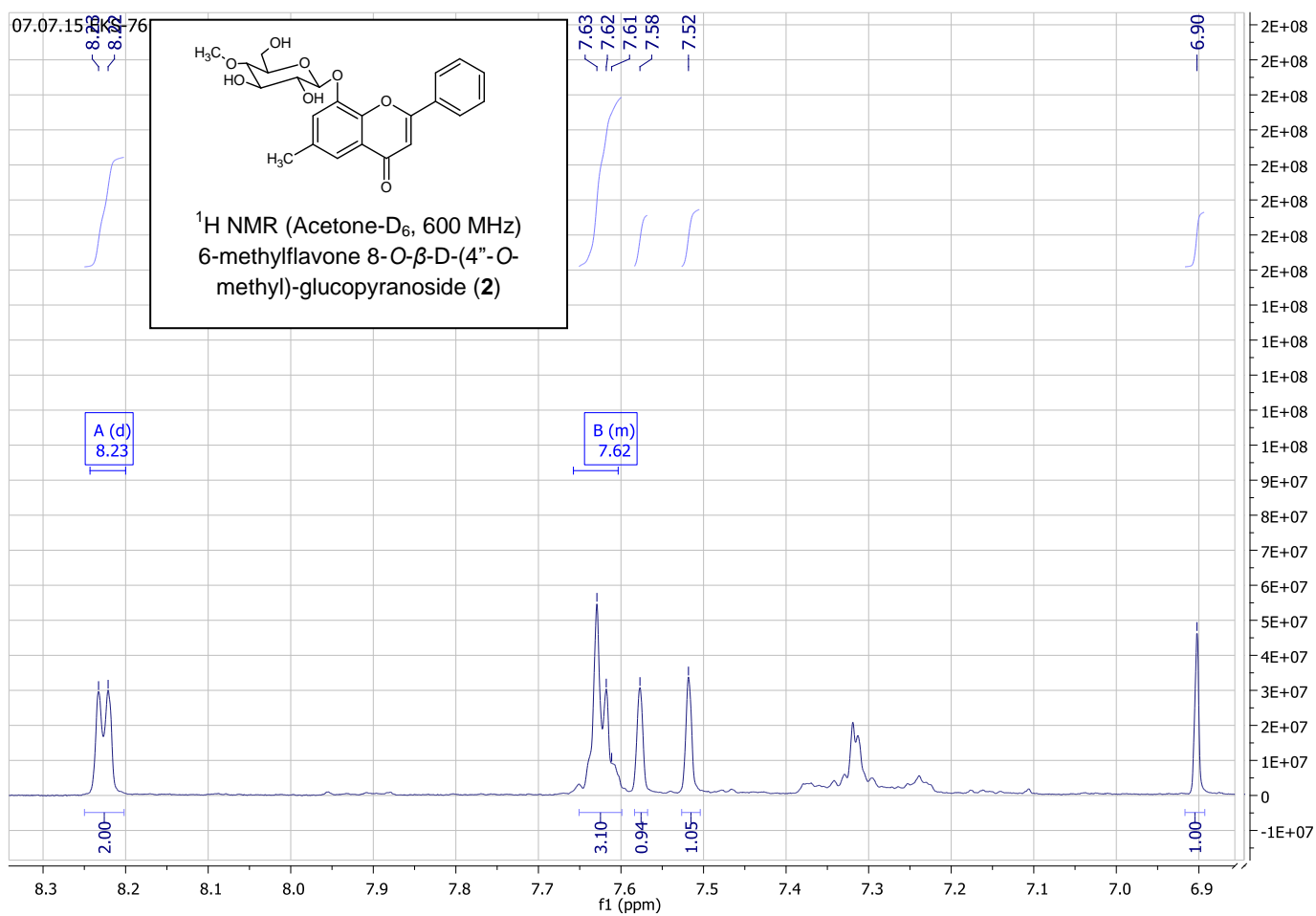

S8 Fig. <sup>1</sup>H NMR of 6-methylflavone 8-O- $\beta$ -D-(4''-O-methyl)-glucopyranoside (2) (Acetone-D<sub>6</sub>, 600 MHz).

Supplement: S8 Fig — (PDF) [file pone.0184885.s008.pdf]

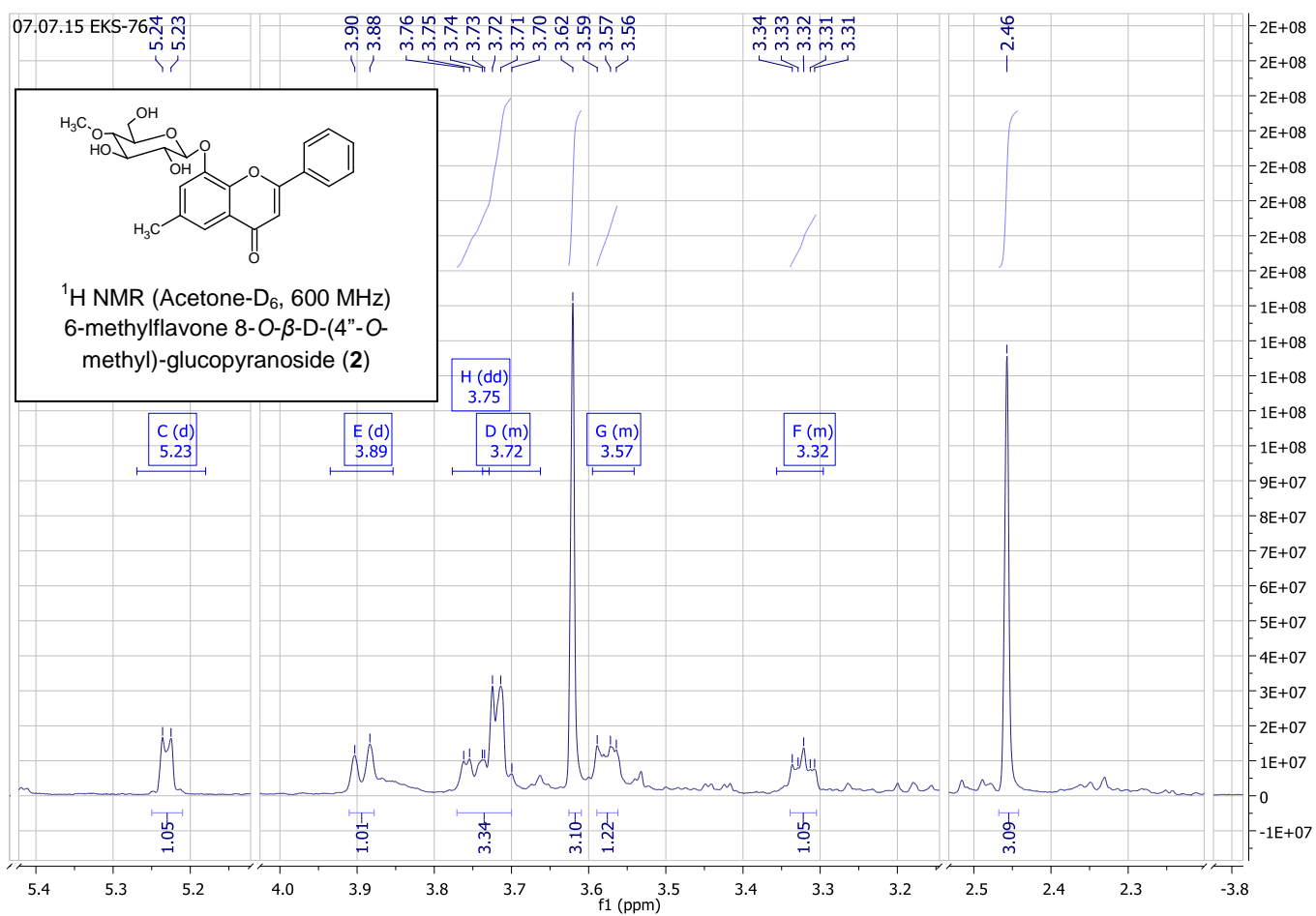

S9 Fig. <sup>1</sup>H NMR of 6-methylflavone 8-O-β-D-(4''-O-methyl)-glucopyranoside (2) (Acetone-D<sub>6</sub>, 600 MHz).

Supplement: S9 Fig — (PDF) [file pone.0184885.s009.pdf]

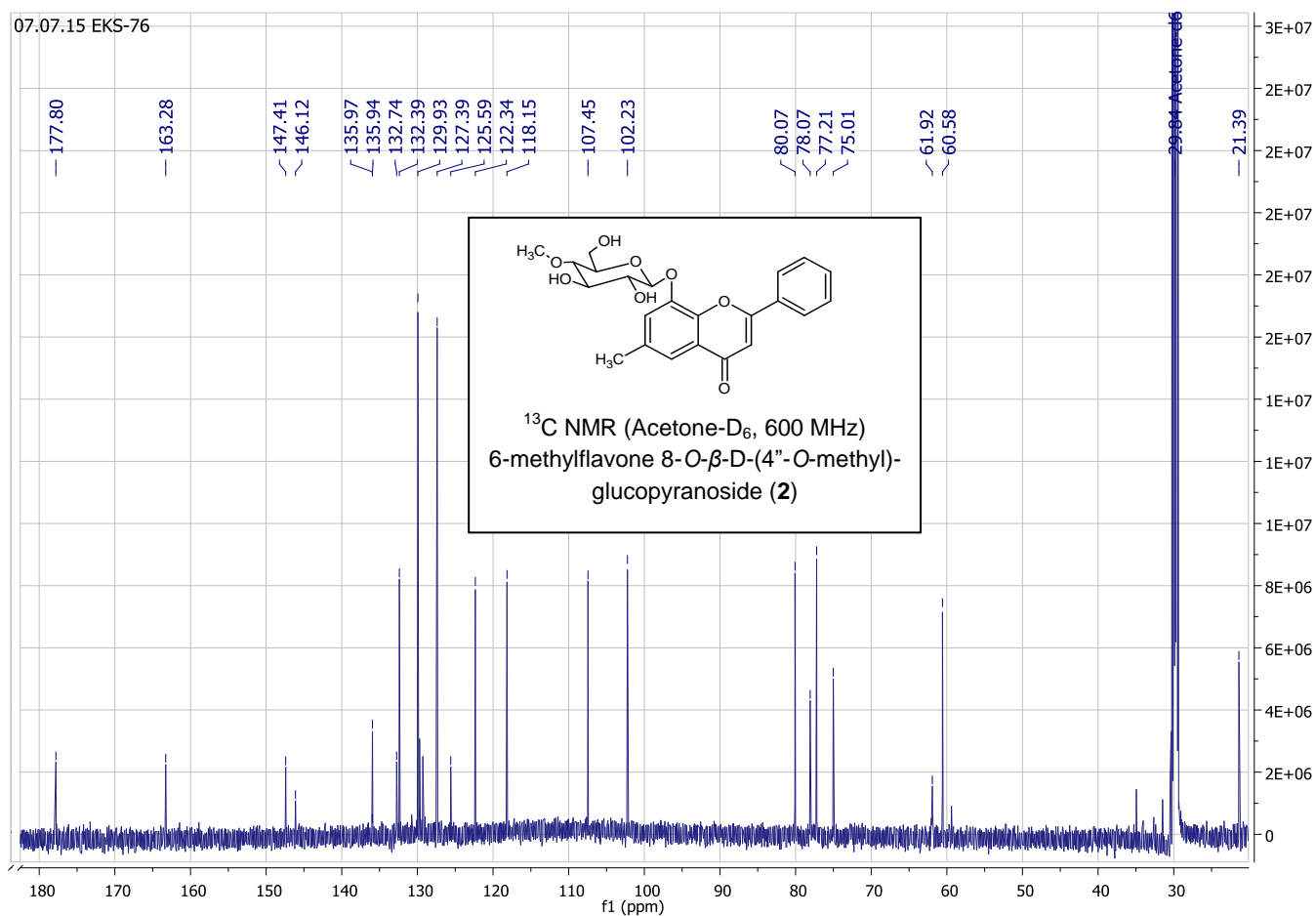

S10 Fig.  $^{13}\text{C}$  NMR of 6-methylflavone 8-O- $\beta$ -D-(4''-O-methyl)-glucopyranoside (2) (Acetone- $\text{D}_6$ , 600 MHz).

Supplement: S10 Fig — (PDF) [file pone.0184885.s010.pdf]

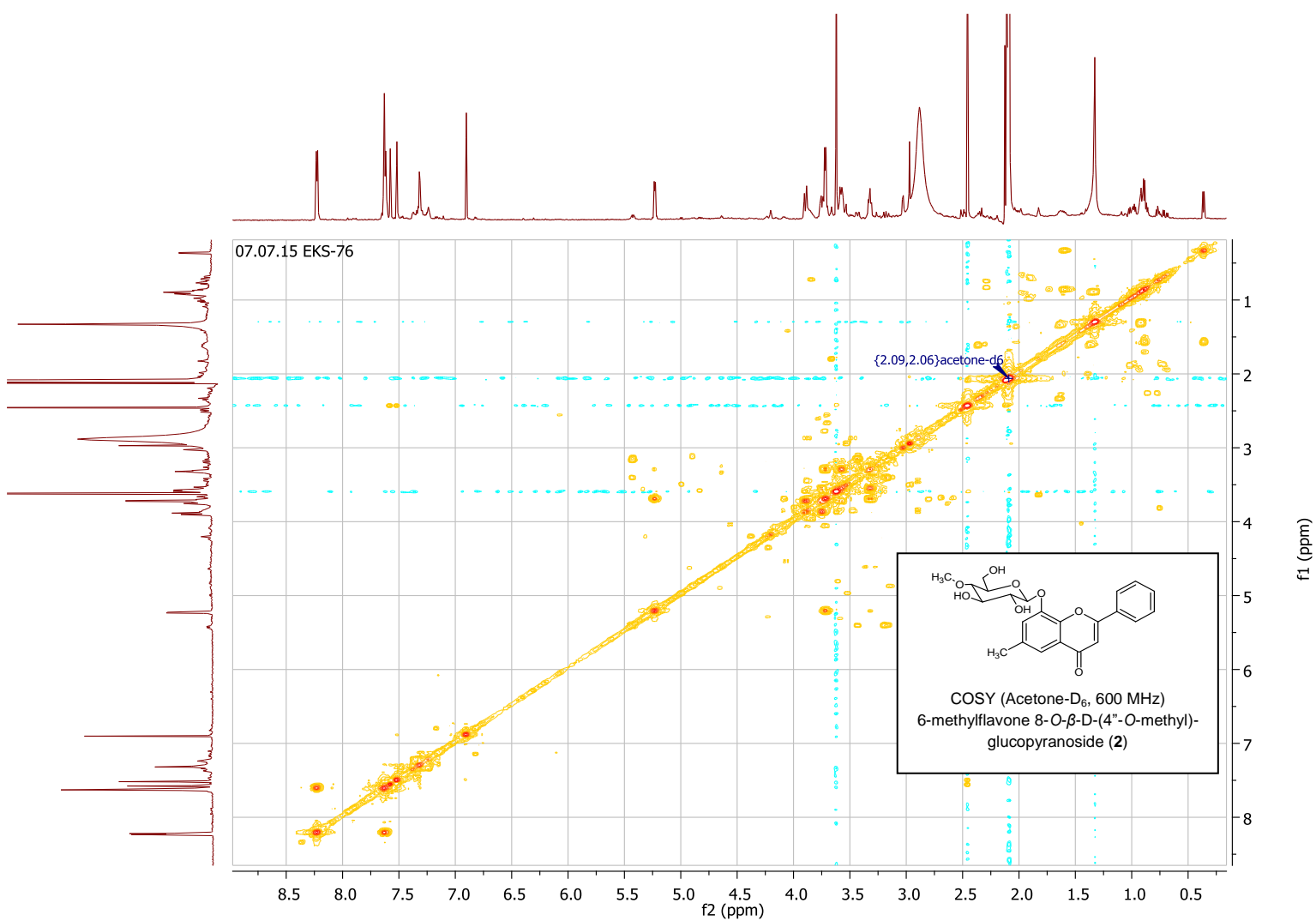

**S11 Fig. COSY NMR of 6-methylflavone 8-O-β-D-(4''-O-methyl)-glucopyranoside (2) (Acetone-D<sub>6</sub>, 600 MHz).**

Supplement: S11 Fig — (PDF) [file pone.0184885.s011.pdf]

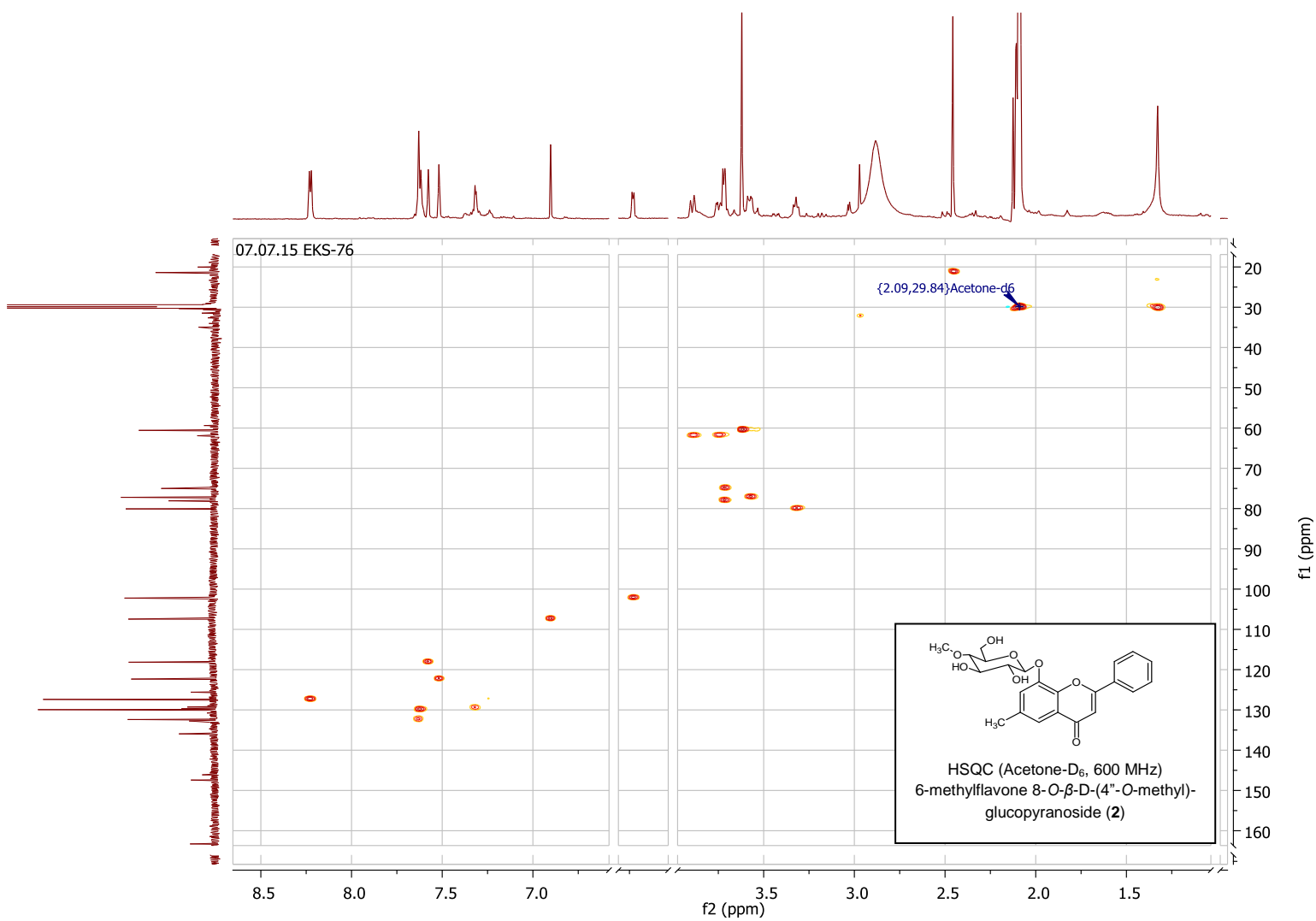

**S12 Fig. HSQC NMR of 6-methylflavone 8-O-β-D-(4''-O-methyl)-glucopyranoside (**2**) (Acetone-D<sub>6</sub>, 600 MHz).**

Supplement: S12 Fig — (PDF) [file pone.0184885.s012.pdf]

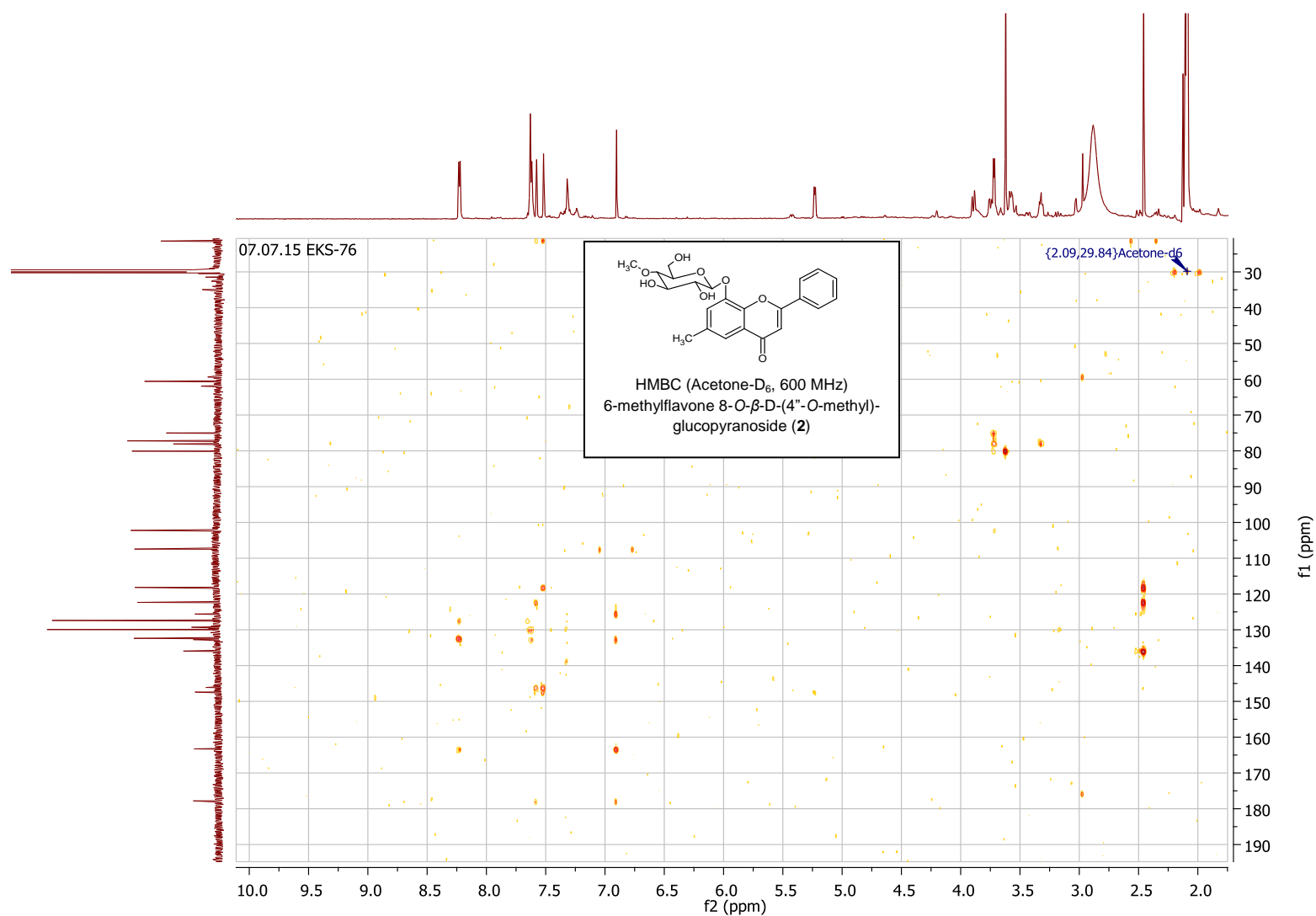

**S13 Fig. HMBC NMR of 6-methylflavone 8-O-β-D-(4''-O-methyl)-glucopyranoside (2) (Acetone-D<sub>6</sub>, 600 MHz).**

Supplement: S13 Fig — (PDF) [file pone.0184885.s013.pdf]

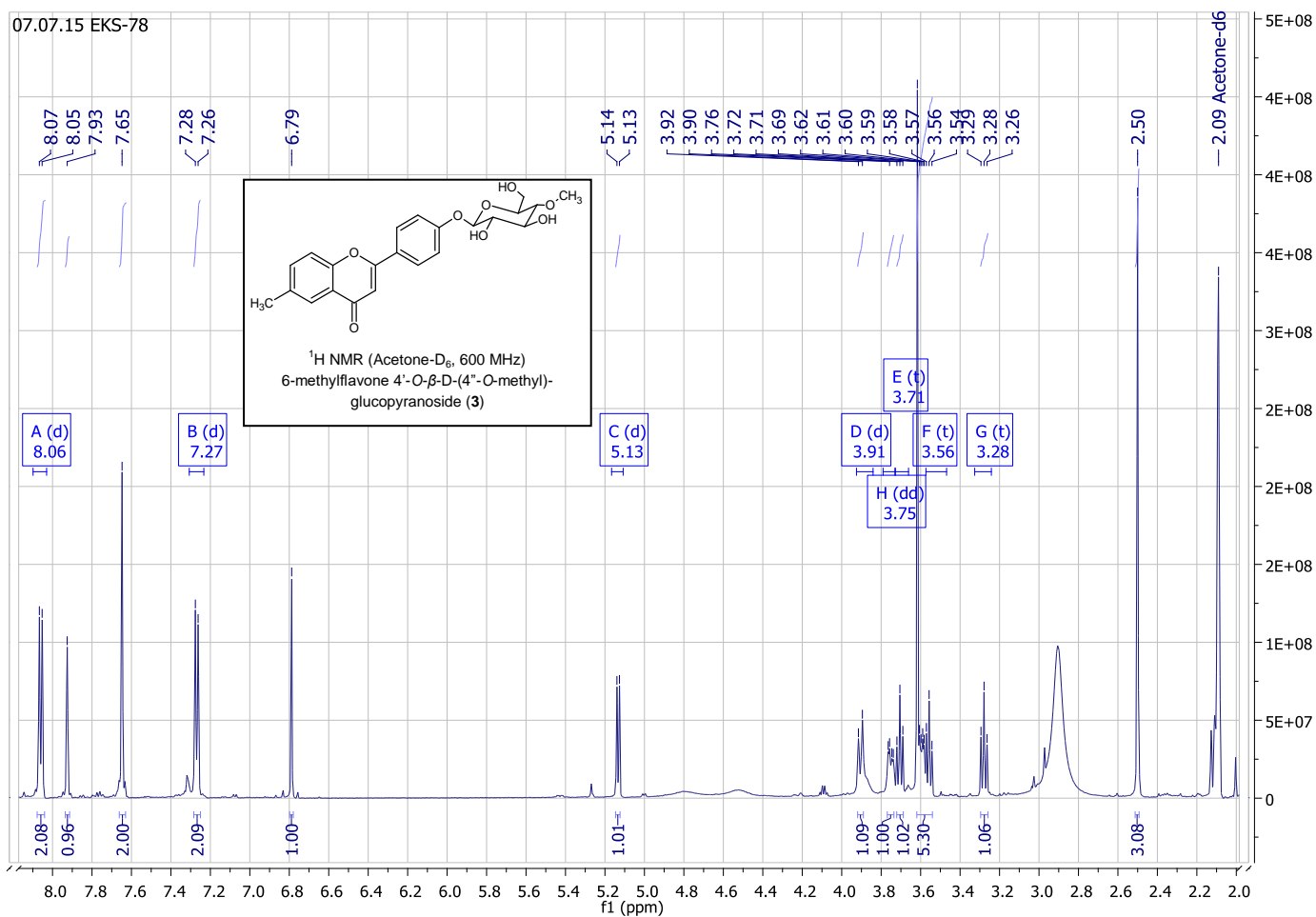

**S14 Fig.  $^1\text{H}$  NMR of 6-methylflavone 4'-O- $\beta$ -D-(4''-O-methyl)-glucopyranoside (3) (Acetone- $\text{D}_6$ , 600 MHz).**

Supplement: S14 Fig — (PDF) [file pone.0184885.s014.pdf]

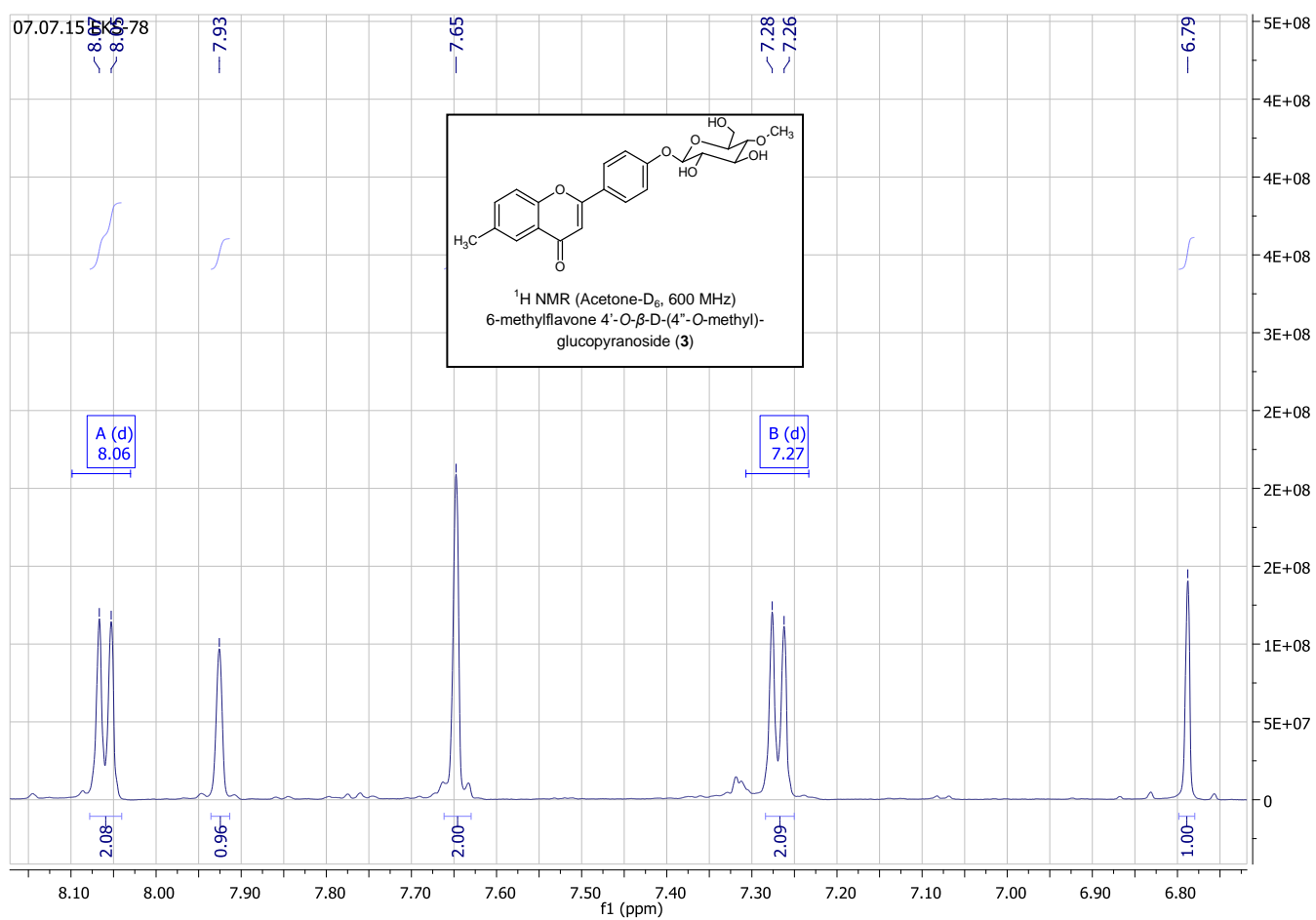

S15 Fig. <sup>1</sup>H NMR of 6-methylflavone 4'-O- $\beta$ -D-(4''-O-methyl)-glucopyranoside (3) (Acetone-D<sub>6</sub>, 600 MHz).

Supplement: S15 Fig — (PDF) [file pone.0184885.s015.pdf]

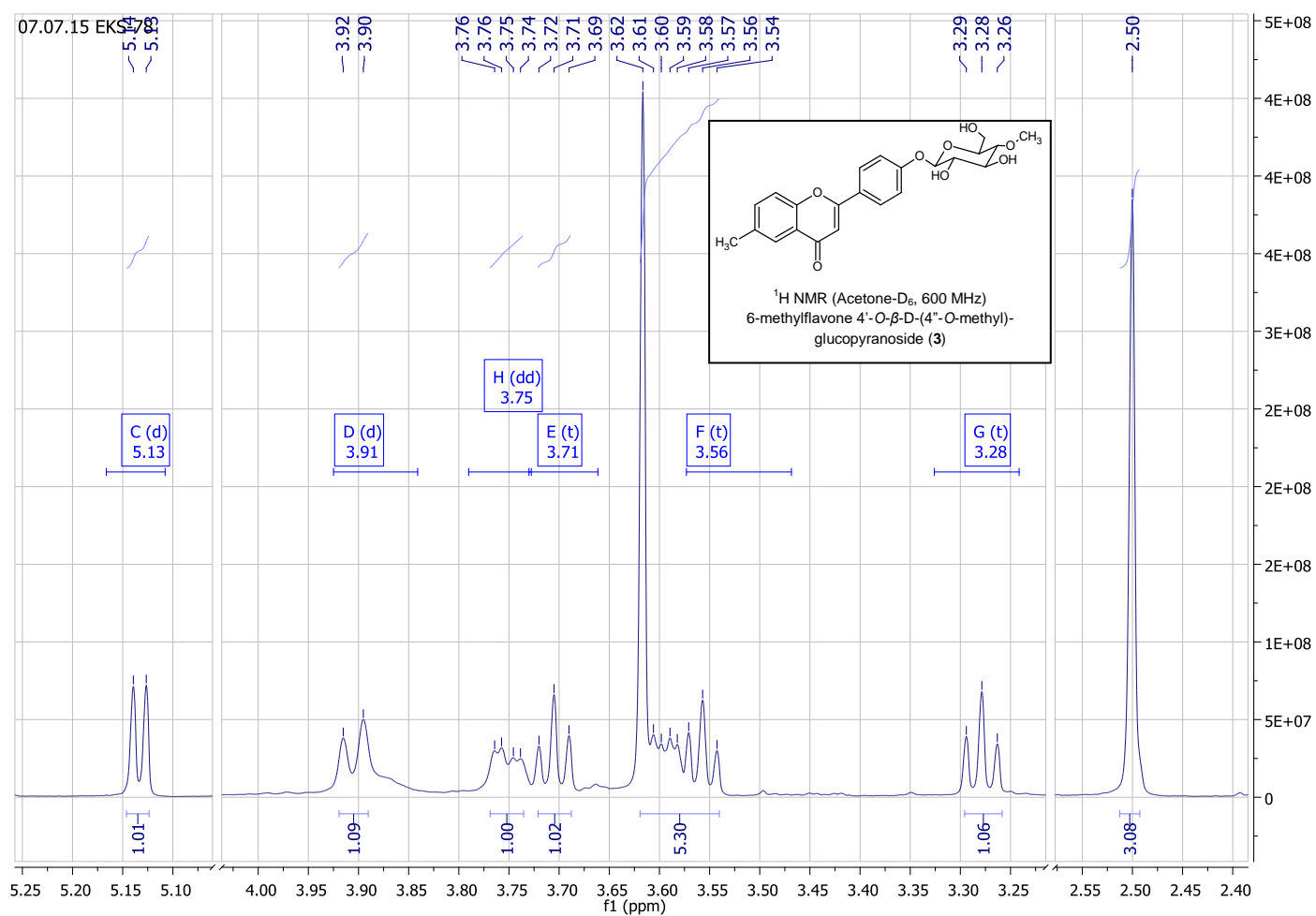

**S16 Fig.  $^1\text{H}$  NMR of 6-methylflavone 4'-O- $\beta$ -D-(4''-O-methyl)-glucopyranoside (3) (Acetone- $\text{D}_6$ , 600 MHz).**

Supplement: S16 Fig — (PDF) [file pone.0184885.s016.pdf]

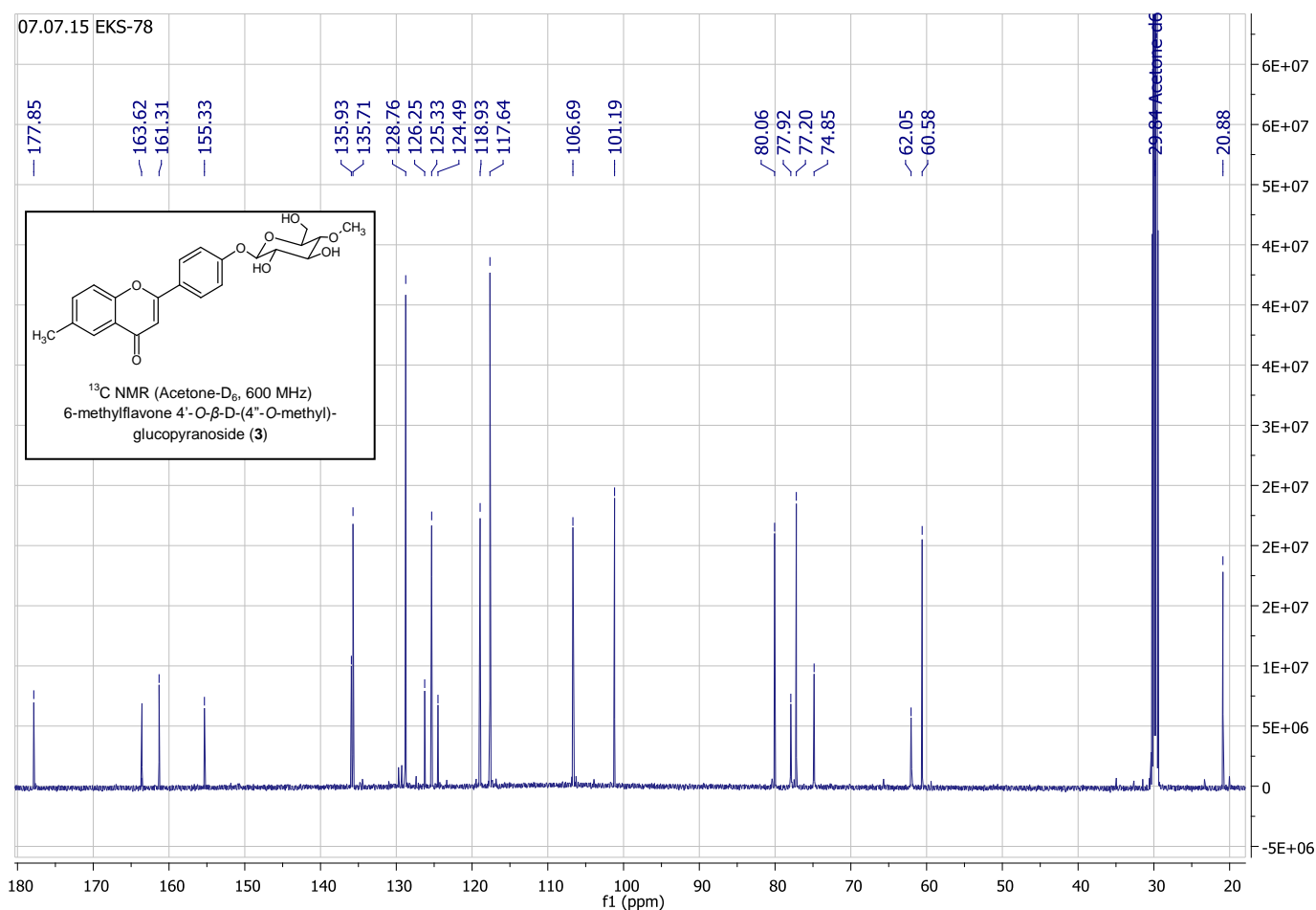

**S17 Fig. <sup>13</sup>C NMR of 6-methylflavone 4'-O-β-D-(4''-O-methyl)-glucopyranoside (3) (Acetone-D<sub>6</sub>, 600 MHz).**

Supplement: S17 Fig — (PDF) [file pone.0184885.s017.pdf]

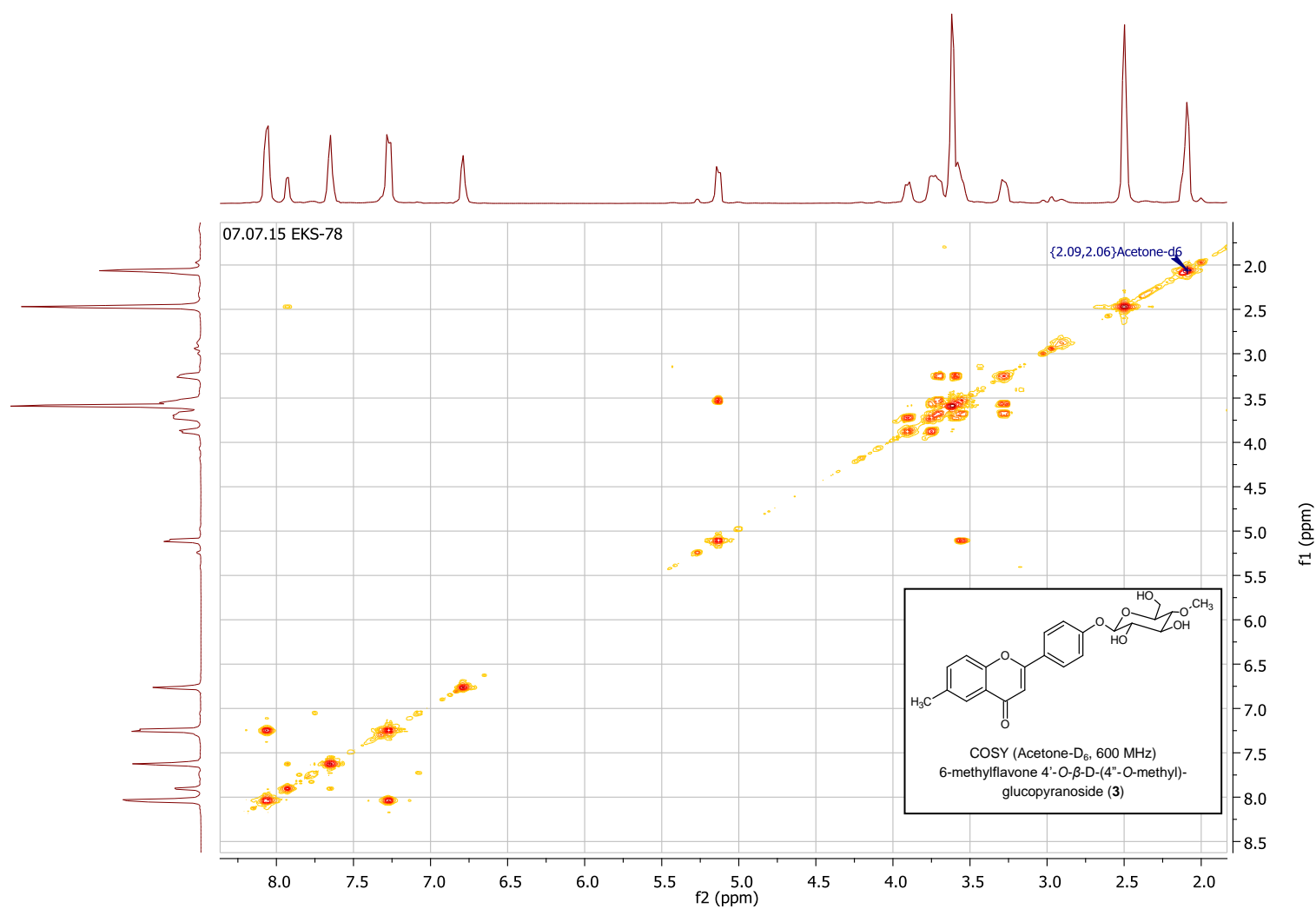

**S18 Fig. COSY NMR of 6-methylflavone 4'-O-β-D-(4''-O-methyl)-glucopyranoside (3) (Acetone-D<sub>6</sub>, 600 MHz).**

Supplement: S18 Fig — (PDF) [file pone.0184885.s018.pdf]

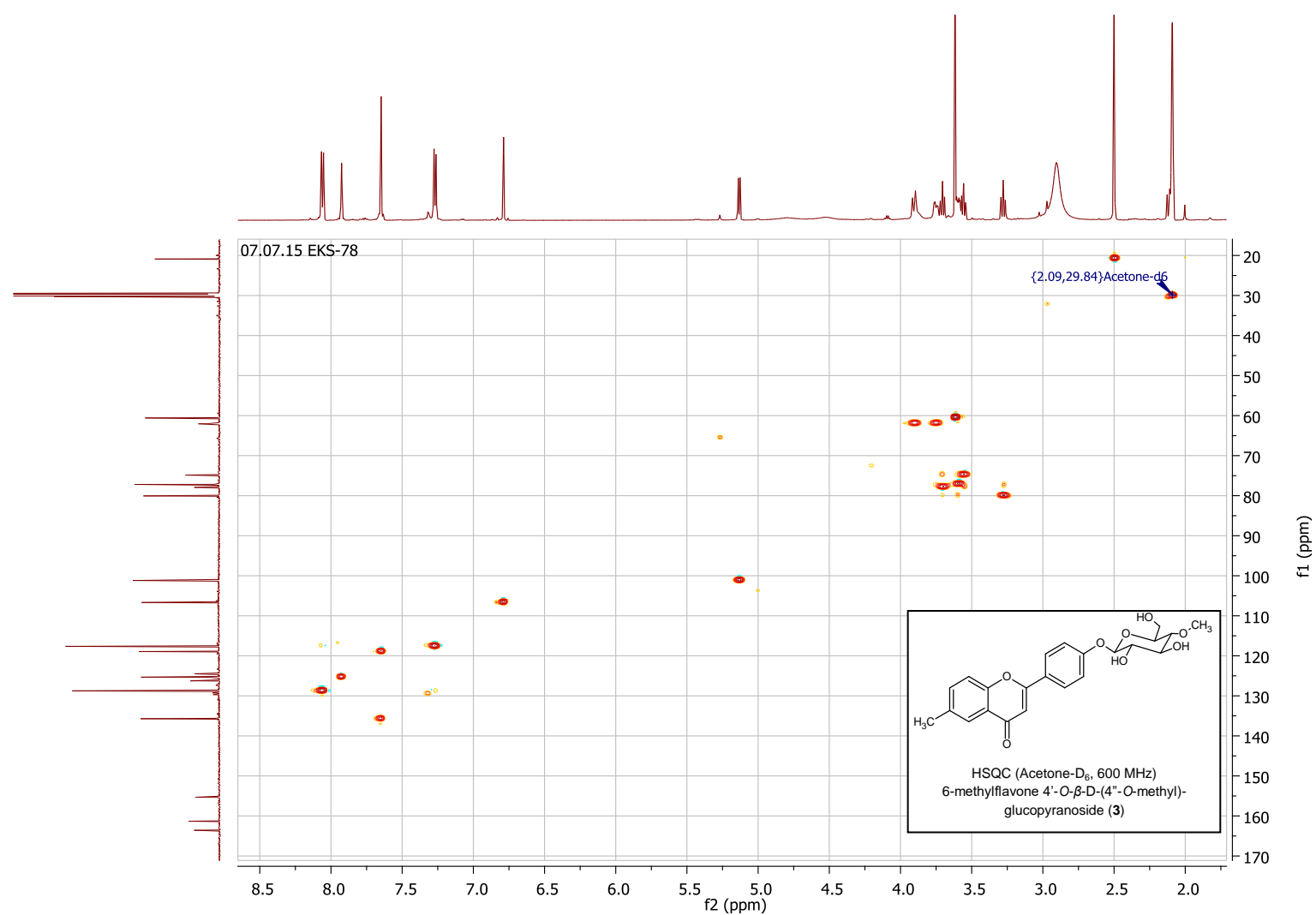

**S19 Fig. HSQC NMR of 6-methylflavone 4'-O-β-D-(4''-O-methyl)-glucopyranoside (3) (Acetone-D<sub>6</sub>, 600 MHz).**

Supplement: S19 Fig — (PDF) [file pone.0184885.s019.pdf]

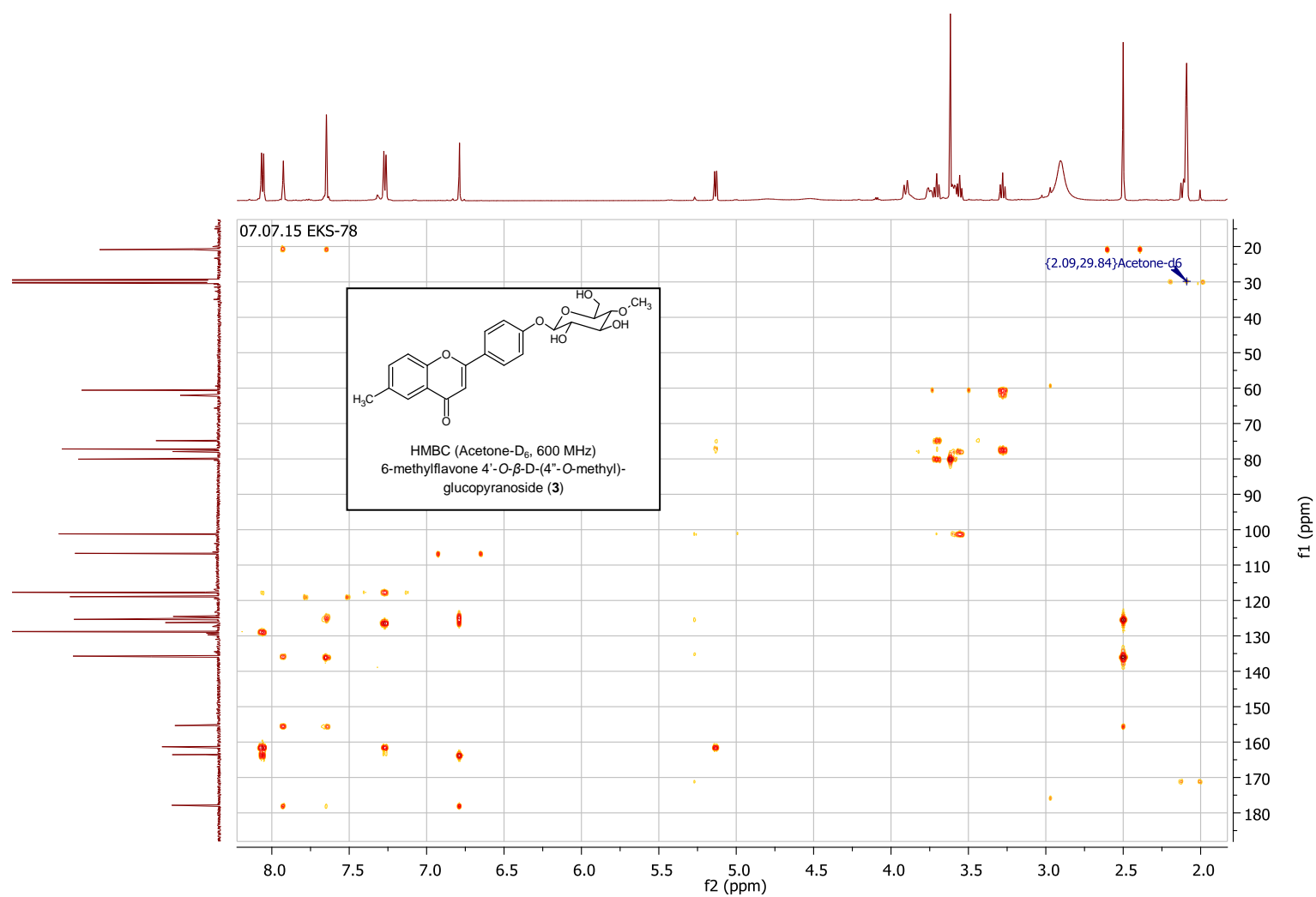

**S20 Fig. HMBC NMR of 6-methylflavone 4'-O-β-D-(4"-O-methyl)-glucopyranoside (3) (Acetone-D<sub>6</sub>, 600 MHz).**

Supplement: S20 Fig — (PDF) [file pone.0184885.s020.pdf]
